# Caper (*Capparis spinosa* L.): An Updated Review on Its Phytochemistry, Nutritional Value, Traditional Uses, and Therapeutic Potential

**DOI:** 10.3389/fphar.2022.878749

**Published:** 2022-07-22

**Authors:** Hassan Annaz, Yaya Sane, Gabin Thierry M. Bitchagno, Widad Ben Bakrim, Badreddine Drissi, Ismail Mahdi, Mustapha El Bouhssini, Mansour Sobeh

**Affiliations:** ^1^ AgrobioSciences Research, Mohammed VI Polytechnic University, Ben-Guerir, Morocco; ^2^ African Sustainable Agriculture Research Institute (ASARI), Mohammed VI Polytechnic University (UM6P), Laayoune, Morocco

**Keywords:** capparaceae, antidiabetic, hepatoprotective, flavonoids, glucosinolates, indoles, caper bush, flinders rose

## Abstract

Caper (*Capparis spinosa* L.) is a perennial shrub of the family Capparaceae, endemic to circum-Mediterranean countries. Caper carries a renowned nutritional value, especially in terms of vitamins and antioxidants related to the occurrence of flavonoids, alkaloids, and glucosinolates as main secondary metabolites. Caper extracts have also shown to display antibacterial, antifungal, analgesic, antitumor, hepatoprotective, antioxidant, anti-inflammatory, and neuroprotective effects which correlate the uses of the plant in folk medicine against both metabolic and infectious diseases. The present review aims to provide exhaustive phytochemistry and pharmacological properties survey on Caper constituents. Attention has also been given to the nutritional values and traditional uses of main organs to pinpoint research gaps for future investigations on the plant.

## Introduction

Spices constitute one of the valuable ingredients for making dishes worldwide. The spread of spices is related to the diversity and cultural groups around the world leading to versatile dishes (Dinesha and Chikkanna, 2014). Spices are hot, salty, sweet, or sharp inducing secretion of saliva, promoting digestion, preventing cold, and avoiding nausea and vomiting (Kuete et al., 2011). Most of these ingredients are also used by traditional healers to cure several diseases including cancer, microbial infections, and gastrointestinal diseases (Sharma and Sharma, 2012).

Caper (*Capparis spinosa* L*.*) is a perennial spiny shrub of the family of Capparaceae with fleshy leaves and big white to pinkish-white flowers. Caper is salt tolerant and resistant to drought, grows up to 4 m in height in warm and dry weather, and has extensive root systems which can extend up to 6–10 m (Manikandaselvi and Brindha, 2014; Manikandaselvi et al., 2016). The plant is native to the Mediterranean basin but is distributed around Southern Europe, the Northern and Eastern Africa including Madagascar, Southwestern and Central Asia, Indonesia, Australia, Papua New Guinea, and Oceania (Rivera et al., 2003; Nabavi et al., 2016). Caper is plesiomorphic supporting results of studies which reported the polymorphic aspects of the plant and the heterogeneity of its morphological characters. Accordingly, *C. spinosa* are found under 22 variety names when searched in the database (Fici, 2014; Fici, 2015).

Caper is of great interest both as food additives and complementary drugs. The literature abounds with more than 11 reviews on the plant emphasizing the nutraceutical merits, biological effects, or chemical and pharmacological wealth (Mingzhang et al., 1994; Ji et al., 2006; Geng et al., 2007; Xie et al., 2007; Li et al., 2008; Satyanarayana et al., 2008; Yang et al., 2008; Zhao et al., 2014; Manikandaselvi et al., 2016; Rahnavard and Razavi, 2017; Vahid et al., 2017; Zhang and Ma, 2018). These research surveys are not less than 5 years old and most of them are not accessible though they are from Chinese research journals (engines). The present review aimed to provide a detailed exploration of the chemistry and pharmacology of Caper to pinpoint research gaps for future investigations. Data collected in the frame of this work were generated by common research engines such as Web of Science, SciFinder-n, PubMed, ScienceDirect, and Scopus, when entering the references “Caper”, “*Capparis spinosa*” and refining with keywords “chemistry”, “biological”, “antioxidant”, and “anticancer”. A total of 1612 research items were examined out of which 215 fall into the scope of the review, thus, constituting the baseline of the present survey.

## Summary of Bibliometric Analysis of Caper Research

**FIGURE 1:**
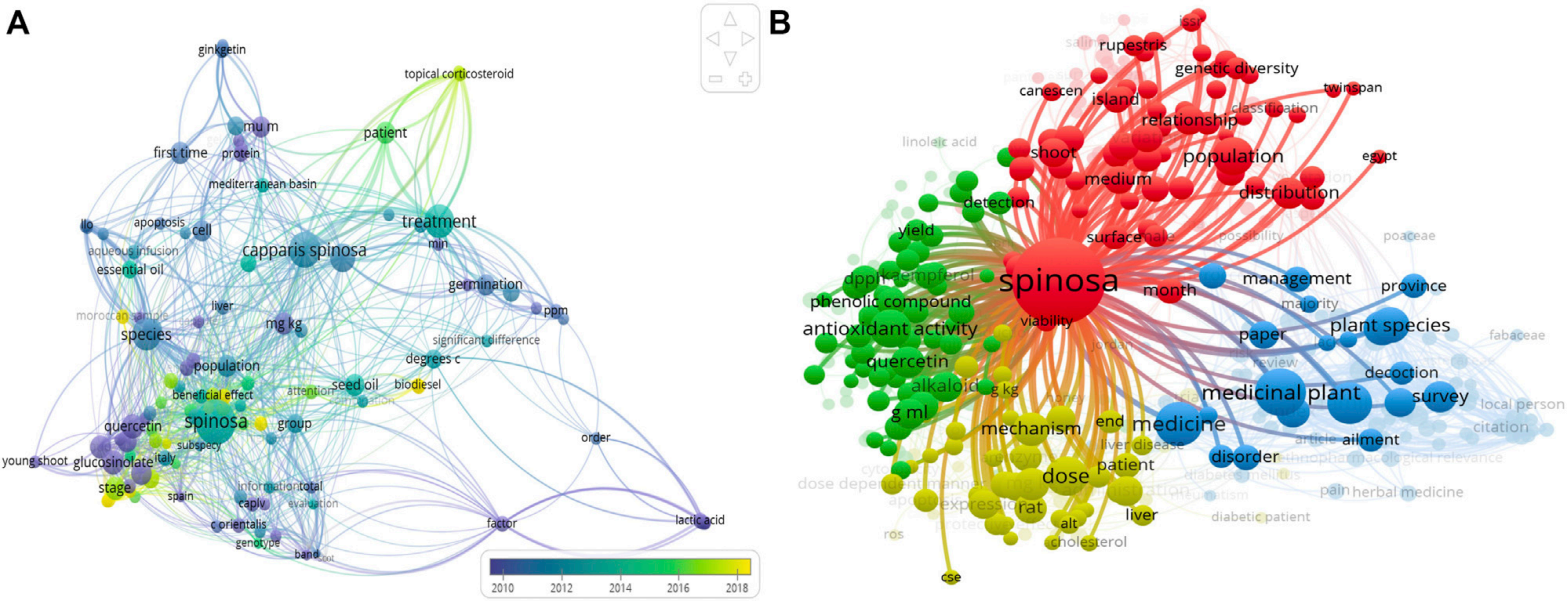
**(A)** Visualization of topic areas of Caper research using overlay visualization. **(B)** Term map generated from all keyword’s fields on Caper and representing the different research themes that defined different clusters. Cluster 1 is colored in red on the term map and represents the botanical and geographical distribution of the plant. This cluster represents research publications on the relationship between the genetic variation and distribution of the plant in different areas. Cluster 2 is colored in green and represents the antioxidant activity of Caper and its correlation with compounds, especially quercetin and kaempferol. Cluster 3 is colored in blue on the map. This cluster represents the ethnopharmacological properties of aper in traditional medicine*.* The different uses depend on the local zones of distribution. Cluster 4 is colored in yellow. The cluster is broadly classified as publications linked with the *in vivo* activity of the plant related to liver disease and diabetes, with the mechanism of the plant on these human diseases.

A total of 835 documents were retrieved from the Scopus database between 2000 and 2021. Most of the documents were original research articles, followed by reviews and other types of documents comprising proceedings papers, book reviews, and meeting abstracts. Noteworthy, there was a gradual rise in annual output from the early 2010s and the highest number of articles were recorded in the United States (Guesmi et al., 2021), Italy (Hameed et al., 2021), China (Mickymaray and Al Aboody, 2019), Turkey (Gull et al., 2015), Iran (Bakr et al., 2016) and Spain (Fu et al., 2008). Furthermore, the over-visualization of the bibliometric analysis shows the trend from the year related to Caper research (Figure 1A). Using VOSviewer, a term map was also created using the occurrences of relevant words. A total of 1432 words were identified from all keyword fields. With the number of occurrences, different research themes were assigned by different clusters (Figure 1B).

## Taxonomical and Vernacular Names

The taxonomical aspect of *C. spinosa* is characterized by high variability due to different factors such as phenotypical plasticity, hybridization processes, selection of cultivated forms, and eco–geographical differentiation. Two recent taxonomic revisions were carried out by Fici, (2014); Fici, (2015) based on the variation of Caper species over a wide geographical range. The taxonomic revision conducted on the geographical area extending from the Mediterranean to central Asia recognized the *Capparis spinosa* as a single species divided into two subspecies, subsp. *Spinosa*, widespread in habitats distinguished by clays, marls, and evaporates, extended from the Mediterranean eastwards to central Asia and Nepal, and subsp. *rupestris* (Sm.) Nyman (1878: 68), a Steno–Mediterranean element extending to the central Saharian massifs, where the habitat is characterized by rocky outcrops and cliffs (Fici, 2014). The other taxonomic revision was conducted in eastern Africa, Madagascar, southern Asia, Australia, and Oceania where four subspecies were referred to, *himalayensis stat. nov, Nummularia, Cordifolia,* and *Cartilaginea*. (Fici, 2015). The distribution of *C. spinosa* worldwide makes it difficult to list every vernacular name. However, it is obvious to find the plant under popular names like Caper (also known as Mariana caper-bush, Mariana caper) in English; kabar, alaf-e-mar in late Persia region; kabbar in the Arabic language; câprier in French; alcaparro in Spain; cappero in Italy; melada in Malaysia; alcaparras in the Philippines or even himsraa, kaakdaani, and kabara in India (Zohary, 1960; Jacobs, 1964; Tutin et al., 1976; Metcalfe, 2006; Ezzeddine et al., 2007).

## Traditional Uses

Caper drains a long history of ethnomedicinal practices worldwide, each organ is concerned (Table 1) (Eddouks et al., 2002). In Iran, fruits and roots bark are used as diuretics and tonics against malaria and hemorrhoids (Hooper and Field, 1937; Afsharypuor et al., 1998). The leaves of the plant serve as an analgesic, aperients, and depurative in Pakistan (Sher et al., 2012). The whole plant and roots of the plant are employed to relieve paralysis, against rheumatism, toothache, and to kill worms in the ear against coughs (Tlili et al., 2011). The decoction of the root bark or the infusion as the tea of young shoots is applied in China against rheumatism, stomachache, anemia, and to treat dropsy (Miraldi et al., 2001; Feng et al., 2011). Moroccans use the buds or leaves as herbal tea or decoction to alleviate eye infections, gastrointestinal infections, diabetes, and the removal of kidney stones. Caper is also deeply involved in Mediterranean gastronomy, it is used as pickles in salads and sauces, as well as condiments and seasoning spices (Gamble, 1972). In addition, the Caper is used in cosmetics, where roots extract is beneficial in treating rose-colored rashes and capillary weaknesses (Chiej, 1984).

**TABLE 1:** Selected ethnomedicinal uses of Caper.

Region	Part Used	Method of Uses	Ethnomedicinal Uses	Ref
Iran	Root, fruit, and bark	Diuretics and tonics	Malaria and joint disease	Hooper and Field, (1937), Afsharypuor et al. (1998)
Iran	Fruits and roots		Hemorrhoids and gout	
Pakistan	Leaves		Analgesic, anti-hemorrhoid, anti-rheumatic, aperients, deobstruent, depurative, and diuretic	Sher et al. (2012)
India	Buds and roots Leaves		Boils counter-irritant and as a cataplasm in swellings	Tlili et al. (2011)
Egypt	Roots		Kidneys and liver disorders	Tlili et al. (2011)
Romania	Plants		Paralysis	Tlili et al. (2011)
	Roots Bark		Fever, rheumatism, paralysis, toothache and kill worms in the ear against coughs, asthma, and inflammation	
China	Stem leaves, fruits, and roots		Treatment of rheumatoid arthritis and gout	Feng et al. (2011), Miraldi et al. (2001)
	Root bark		Analgesic and carminative agent	Ao et al. (2007), Eddouks et al. (2004)
	Root bark	Decoctions	Treat dropsy, anemia, and rheumatism	
	Root and young shoots	Herbal tea	Rheumatism, stomach, and intestinal disorders	
Saudi Arabia	The root bark	Body tonic utilization, pastes prepared	Swollen joints, skin rashes, and dry skin	
Morocco	Unopened buds, dried fruits	Orally with a glass of water	Eye infections hypertension and diabetic complications	Jouad et al. (2001), Eddouks et al. (2002)
	Buds and leaves	Herbal tea	Cold and related infections	
	Buds and leaves	Decoction	Gastrointestinal infections, diarrhea, and dysentery and useful for the removal of kidney stones	Sher and Alyemeni, (2010)

## Nutraceutical Values of Caper

Caper is known as rich in vitamins and fibers, with a minimal amount of fats and calories (Tlili et al., 2009). The plant is considered a good source of vitamins B1, B3, B6, and B9 and moderate in vitamin E. Caper flowering buds also contain a good amount of vitamins A, C, and K (Tlili et al., 2010; Nabavi et al., 2016; Ulukapi et al., 2016). Minerals also abound in Caper mainly calcium, iron, potassium, phosphorus, magnesium, zinc, and manganese, which play a very important role in maintaining proper metabolic activities (Özcan et al., 2004; Arslan and Ozcan, 2007).

## Chemical Constituent’s Synopsis of Caper

Different organs of Caper have been investigated to clear the plant’s chemical constituents. Overall, as awaited, the fruits of the plant were the most screened organ. Caper produces diverse secondary metabolites including alkaloids, sulfur-containing indoles, flavonoids, furan and pyrrole derivatives, tetraterpenes, phenolic acids, and sterols (Moufid et al., 2015; Zhang and Ma, 2018). Rare primary metabolites like nucleosides and nucleic acids have been highlighted to occur in Caper alongside other classes of compounds but with at most two members each. Benzidane et al. (2020) compared the chemical composition through a comprehensive analytical TLC diagram of both aqueous and methanol extracts from fruits, leaves, roots, flowers, seeds, root bark, and twigs. All in all, the methanol extracts sound more chemically rich than aqueous extracts. In addition, except for the fruit, leaves, and flowers, the other plant organs are chemically poor (Benzidane et al., 2020). This statement was typically confirmed looking at reports in the literature since works concentrated on either fruit, leaves, or flowers of the plant.

## Nucleotides and Nucleic Acids

**FIGURE 2:**
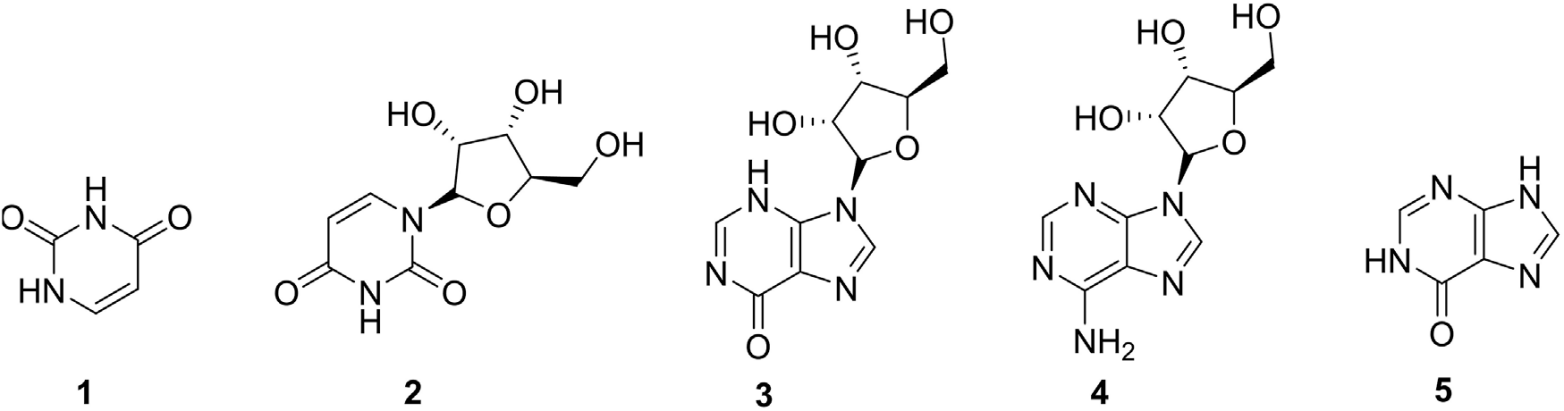
Nucleosides and nucleic acids from *Caper.* **1** = Uracil, **2** = Uridine, **3** = Inosine, **4** = Adenosine, **6** = Hypoxanthine.

The occurrence of nucleosides (**2-4**) and nucleic acids (**1-5**) (Figure 2) are quite comprehensive in the animal kingdom since they intervene in the energy production through ATP formation; however, their presence in the plant kingdom is not common. Uracil (**1**) and adenosine (**3**) are the most abundant members of this group reported in fruits of the plant (Fu et al., 2007; Li et al., 2014). Nucleosides and nucleic acids could be extracted from plants following the total alkaloid extraction method and recovered with *n*-butanol (Fu et al., 2007).

## Alkaloids

**FIGURE 3:**
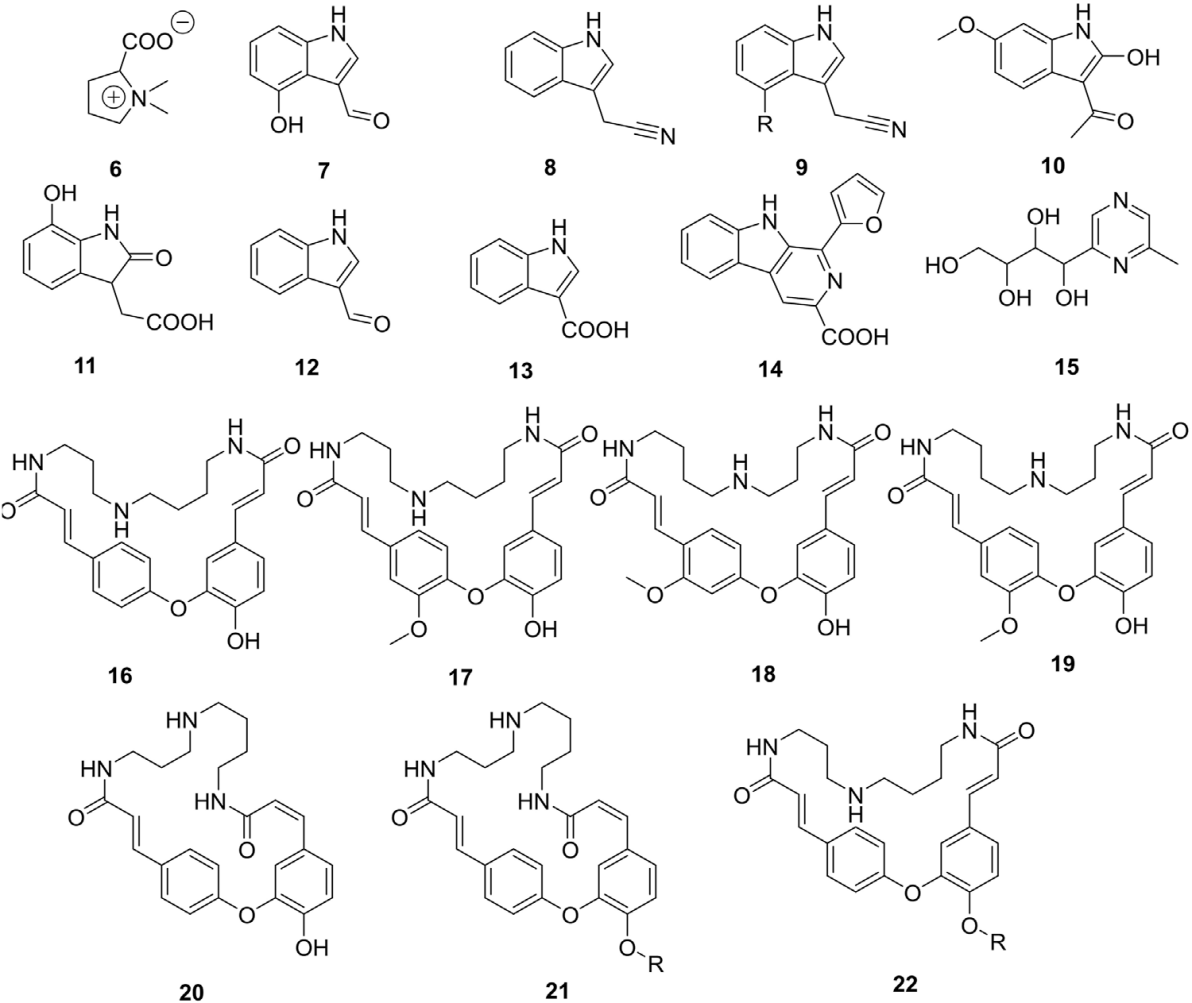
Alkaloids isolated from roots and fruits of Caper. R = *β*-D-glucopyranosyl, **6** = (-)-Stachydrine, **7** = 4-Hydroxy-1*H*-indole-3-carboxaldehyde, **8** = Cappariloside A, **9** = Cappariloside B, **10** = 1-(2-Hydroxy-6-methoxy-1*H*-indol-3-yl)ethanone, **11** = 2,3-Dihydro-7-hydroxy-2-oxo-1*H*-indole-3-acetic acid, **12** = Indole-3-carbaldehyde, **13** = Indole-3-carboxylic acid, **14** = Flazin, **15** = 1-(6-Methyl-2-pyrazinyl)-1,2,3,4-butanetetrol, **16** = Cadabicine, **17** = Isocodonocarpine, **18** = Capparisine, **19** = Codonocarpine, **20** = Capparispine, **21** = Capparispine 26-*O*-*β*-D-glucose, **22** = Cadabicine 26-*O*-*β*-D-glucose.

Alkaloids (**6-22**) (Figure 3) are one of the largest groups of compounds in Caper. They constitute 0.91 and 0.86% of mass material from root bark and fruits, respectively. Alkaloids have not yet been reported in the leaves of the studied plant. Two main classes of alkaloids have been isolated so far, indoles (**7-14**) and spermidines (**16-22**). Spermidines occur almost exclusively in the roots in the yield of 3.5 mg/g of dried material (Khatib et al., 2016), while indoles are abundant in the fruits. The total alkaloid fraction of the fruits retrieved with *n*-butanol afforded the cyanidoindol alkaloid, cappariloside A (**8**), as part of other compounds carried over (Fu et al., 2007). The 6′-glucopyranosyl isomer of compound **8**, cappariloside B (**9**), was rather found in the MeOH extract of dried mature fruits of the plant alongside compound **8** (Çaliş et al., 1999). Both compounds have also been found in the water-soluble fraction of the same plant material as part of the complex mixture of compounds obtained (Çalış et al., 2002). One of the scarce amino acids also abundant in every organ of the plant namely stachydrine (**6**), has been always isolated in its zwitterion form. Mukhamedova et al. (1969) and Sadykov et al. (1981) evaluated by HPLC/DAD the yield of stachydrine to 87.43% of the total alkaloid extract also quantified to 7.4% of the roots of the plant dried material (Sadykov et al., 1981); (Mukhamedova et al., 1969); (Fu et al., 2008).

## Glucosinolates and Other Sulfur-Containing Compounds

**FIGURE 4:**
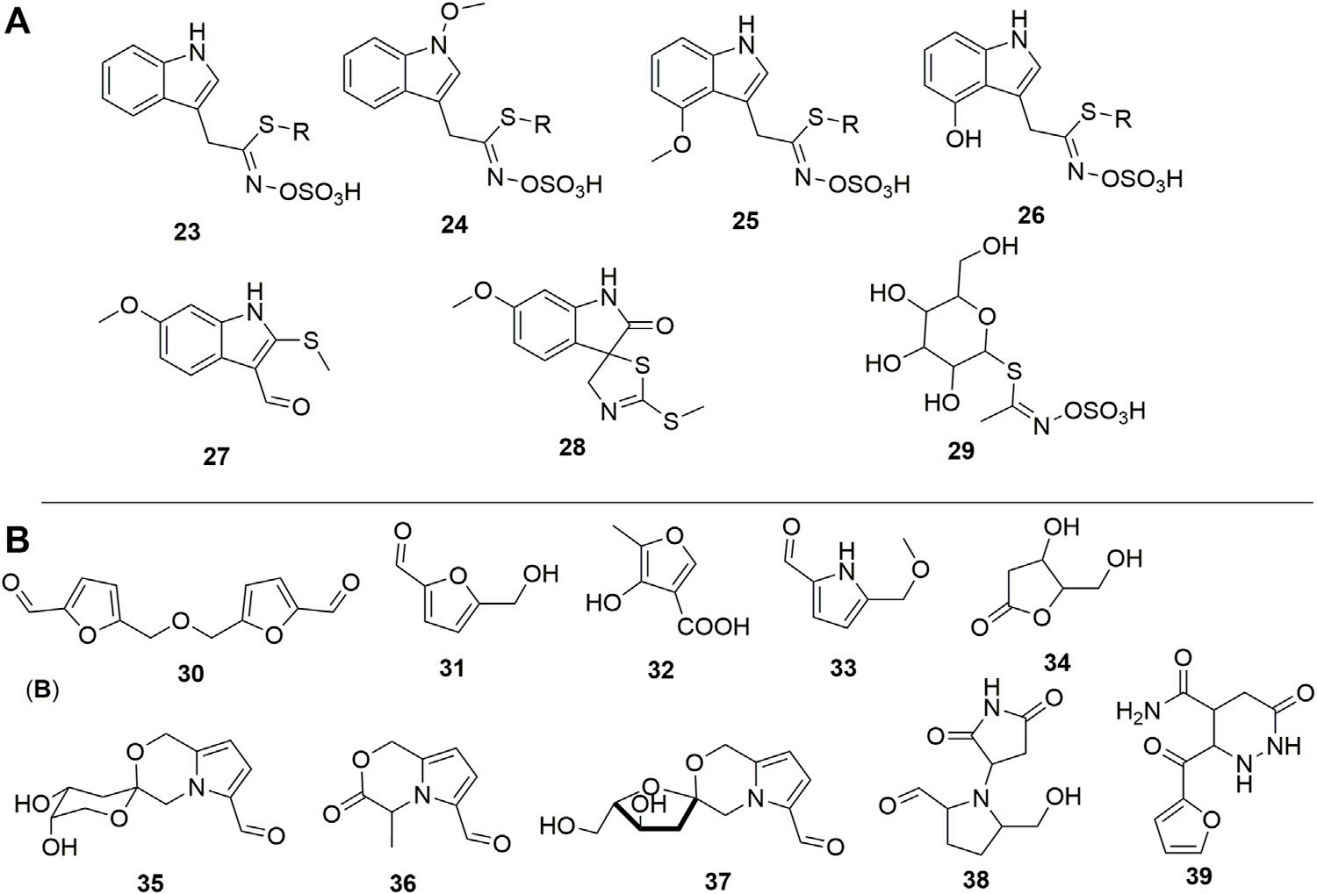
**(A)** Sulfur-containing compounds from Caper. **(B)** Furan and pyrrole analogues reported in Caper. R = *β*-D-glucopyranosyl, **23** = Glucobrassicin, **24** = Neoglucobrassicin, **25** = 4-Methoxyglucobrassicin, **26** = 4-Hydroxyglucobrassicin, **27** = 6-Methoxy-2-(methylthio)-1*H*-indole-3-carboxaldehyde, **28** = (3*S*)-(-)-6-Methoxy-2'-(methylthio)spiro[3*H*-indole-3,5′(4′*H*)-thiazol]-2(1*H*)-one, **29** = Glucocapparin, **30** = 5,5'-[Oxybis(methylene)]bis[2-furancarboxaldehyde], **31** = 5-(Hydroxymethyl)furfural, **32** = 4-Hydroxy-5-methyl-3-furancarboxylic acid, **33** = 5-(Methoxymethyl)-1*H*-pyrrole-2-carbaldehyde, **34** = 3,4,5-trihydroxypentanoic acid *γ*-lactone, **35** = Capparisine B, **36** = 2-(5-hydroxymethyl-2-formylpyrrol-1-yl) propionic acid lactone, **37** = Capparisine A, **38** = *N*-(30-maleimidy1)-5-hydroxymethyl-2-pyrrole formaldehyde, **39** = Capparisine C.

Other alkaloids from indole-type but containing sulfur abundant in the plant are termed glucosinolates (Figure 4A). In this group, sulfur occurs either as a thiol group (**27-28**) or as part of the sulfate fragment. The later mentioned moiety could be essential in the standardization of either an extract, fraction, or pure compounds as it helps to increase the bioavailability of the drugs. Schraudolf (1988) was the first to highlight the occurrence of glucosinolates in the roots with the isolation of compounds (**23-26**) (Schraudolf, 1989). Glucocapparin is one of the main representatives of glucosinolates in Caper (**29**) (Meiliwan et al., 2008; Jiménez-López et al., 2018), and methyl-isothiocyanate and benzyl-isothiocyanate are the main compounds in the series of isothiocyanate derivatives from the fruits (Romeo et al., 2007). They have not been isolated from any plant organ yet, rather the indole derivative (**28**) has been isolated with an integrated isocyanate moiety to the core indole (Zhou et al., 2010).

## Furans and Pyrroles

Furan and pyrrole derivatives (**30-39**), (Figure 4B) constitute another most important group of compounds of Caper. Compounds **30** and **31** are the most representative of this group in the plant since they have been highlighted by many authors (Li et al., 2014; Hu et al., 2017). The differential extraction of the ethanol extract of dried fruits with petroleum ether and ethyl acetate in water yields pyrrole derivatives capparisines A (**35**), B (**37**), and C (**39**), 2-(5-hydroxymethyl-2-formylpyrrol-1-yl) propionic acid lactone (**36**) and *N*-(30-maleimidy1)-5-hydroxymethyl-2-pyrrole formaldehyde (**38**) (Yang et al., 2010a). This is also the unique report of the occurrence of these compounds in Caper.

## Flavonoids

**FIGURE 5:**
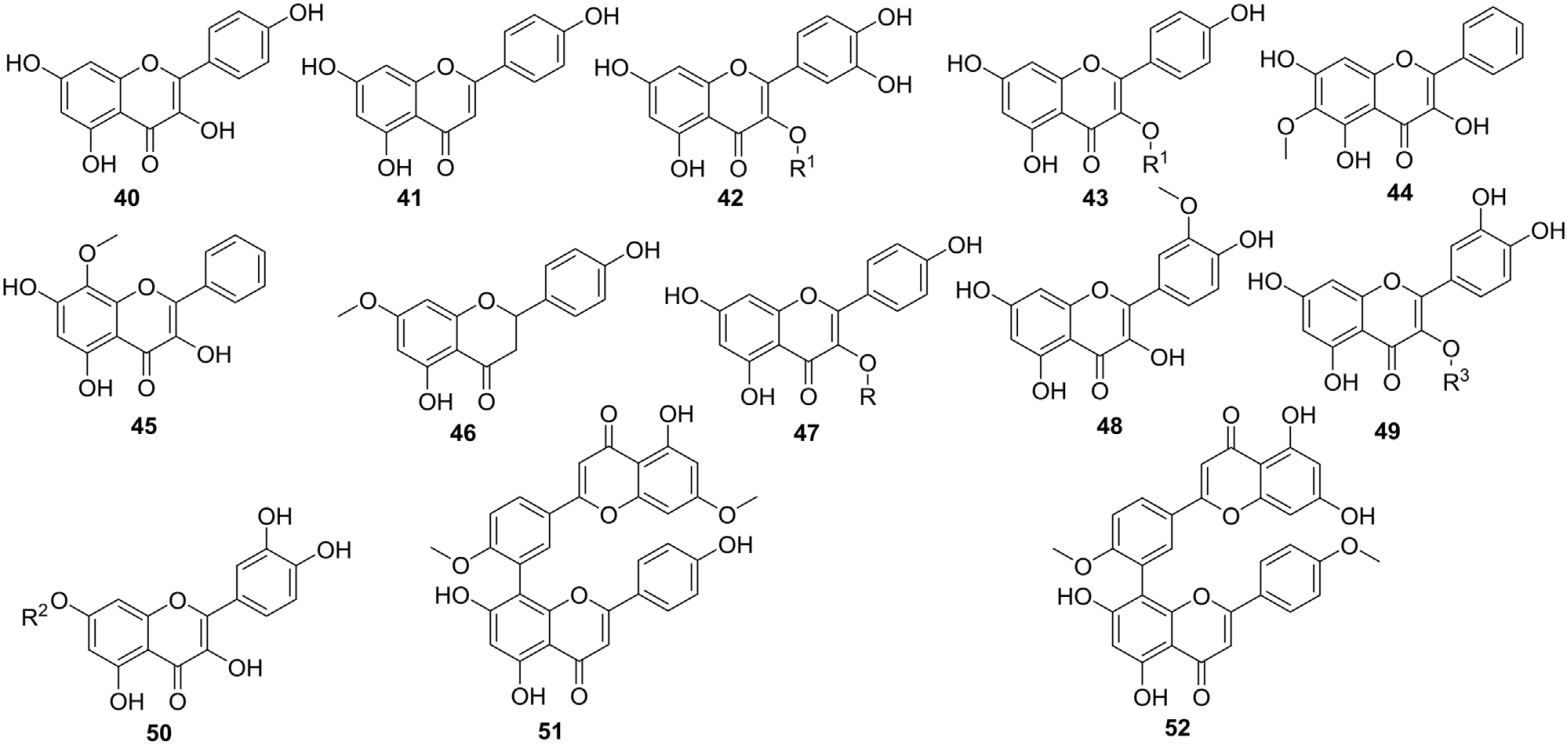
Flavonoids isolated in Caper organs. R = *β*-D-glucopyranosyl, R^1^ = rutinosyl, R^2^ = β-D-glucorhamnoside, R^3^ = 6‘-*O*-rutinosyl-*O*-*β*-D-glucoside, **40** = Kampferol, **41** = Apigenin, **42** = Rutin, **43** = kaempferol 3-*O*-rutinoside, **44** = Oroxylin A, **45** = Wogonin, **46** = Sakuranetin, **47** = Astragalin, **48** = quercetin 3-*O*-[6‴-*α*-L-rhamnosyl-6″-*β*-D-glucosyl]-*β*-D-glucoside, **49** = Isorhamnetin, **50** = Quercetin 7-*O*-*β*-D-glucorhamnoside, **51** = Ginkgetin, **52** = Isoginkgetin.

The total flavonoid content of Caper ranged from 4.71 to 72.79 mg equivalent to quercetin per Gram of dried material (QE/g DR) including compounds **40**–**52** (Figure 5) (Tlili et al., 2015). Benzidane et al. (2020) studied the occurrence of rutin, quercetin, catechin, and gallic acid in both methanol and aqueous extracts of Caper organs. The methanol extract of the leaves contained rutin in a larger amount compared to the other extracts. However, the methanol extracts of fruits and flowers contained rutin with a similar yield (Benzidane et al., 2020). Ramezani et al. (2008) and Musallam et al. (2012) have come to the same conclusion, assessing the amount of rutin in hydroalcoholic extracts of these three main organs of Caper (Ramezani et al., 2008; Musallam et al., 2012). Rutin was found in large amounts in leaves (62 mg/100 g) followed by flowers (44 mg/100 g) and fruits (6 mg/100 g). Comparing microwave, Soxhlet, and decoction methods in the extraction of rutin, Mollica et al. (2019) indicated how effective the Soxhlet extraction method was in the targeted compound compared to the others (Mollica et al., 2019). Likewise, both methanol and aqueous extracts of the fruits showed to contain either catechin or gallic acid since these standards travel with the same frontal report on the plate. Quercetin was almost absent in the extracts examined (Benzidane et al., 2020).

Other methods have been applied to isolate and identify flavonoids in Caper. Results confirm the absence of quercetin in the plant organs. Rather, methoxylated flavonoids (**44-45, 48**) have also been isolated from Caper organs. In addition, a quercetin triglucoside (**49**) has been mentioned to occur in the 80% hydromethanolic extract of the aerial part together with quercetin 3-*O*-*β*-D-glucopyranoside (**47**) (Sharaf et al., 2000). Other flavonoid glycosides, mainly quercetin 7-*O*-*β*-D-glucorhamnoside (**50**), have also been reported from the *n*-butanol fraction of the aerial part of the plant (Artemeva et al., 1981). To date, only two biflavonoids, isoginkgetin, and ginkgetin, amongst polyflavonoids have been reported from the fruits of the studied plant or one of the other organs (Zhou et al., 2011).

## Phenolic Acids and Fatty Acids

The yield of an extract in phenolic acids could be linked to the extraction methods. The maceration method has shown higher levels of phenolic acids and flavonoids compared to ultrasonic-assisted extraction and reflux (Yahia et al., 2020). Quinic, gallic, and protocatechuic acids were the most abundant phenolic acids in the leaf extracts (Yahia et al., 2020). These acids and others which include chlorogenic, *p*-hydroxybenzoic, vanillic, caffeic, syringic, *p*-coumaric, ferulic or rosmarinic acids, and vanillin, protocatechuic aldehyde, and syringaldehyde were not detected in similar research on the fruits of the plant. However, gentisic, sinapic and benzoic acids were identified (Aliyazicioglu et al., 2013). Quinic acid, chlorogenic acid, and *p*-coumaroyl quinic acid were checked by LC-ESI-MS/MS from the aerial part of the plant (Bakr et al., 2016). By all, the total phenolic content (including phenolic acids, flavonoids, and coumarins) of the plant seeds ranged from 1.31 mg gallic acid equivalents per g of dry residue (GAE/g DR) to 8.14 mg GAE/g DR (Tlili et al., 2015). Leaves content in phenolic acids corresponded to 427.27 mg GAE/g DR (Mansour et al., 2016) while fruits carry 1.43 mg GAE/g DR and small buds, 5.97 mg GAE/g DR (Ghafoor et al., 2020). Nonetheless, the content of a plant extract in phenolic compounds could be drastically affected by fermentation (Sonmezdag et al., 2019). For instance, fresh buds of the plant showed to contain 18.43 mg/g DR which drops to 11.98–15.39 mg/g DR in fermented buds (Aksay et al., 2021) (Table 2). Likewise, polyunsaturated fatty acids constituted 50% of accounted fatty oil content (also representing 1.6%) of the plant (Rodrigo et al., 1992). The most abundant in this series is oleic acid (45.82%) followed by linoleic acid (25.37%), palmitic acid (15.93%), palmitoleic acid (4.55%) and stearic acid (4.06%) (Tlili et al., 2009).

**TABLE 2:** A non-exhaustive list of phenolic acids identified in organs of Caper.

Compound names	Organs	Characterization method	References
Gallic acid	Seeds, flower buds, leaves	HPLC-UV	Tlili et al. (2015), Mollica et al. (2019), Yahia et al. (2020)
Protocatechuic acid	Seeds, fruit, leaves	HPLC-UV	Tlili et al. (2015), Yu et al. (2006), Yahia et al. (2020)
Chlorogenic acid	Seeds, flower buds, aerial	HPLC-UV/DAD	Tlili et al. (2015), Mollica et al. (2019), Bakr et al. (2016)
Methyl gallate	Seeds, flower buds	HPLC-UV, GC-MS	Tlili et al. (2015), Romeo et al. (2007)
Gentisic acid	Seeds	HPLC-UV	Tlili et al. (2015)
Ferulic acid			
2-Hydroxyphenylacetic acid
Propyl gallate	Seeds, flower buds	HPLC-UV, GC-MS	Tlili et al. (2015), Romeo et al. (2007)
Methyl 4-hydroxybenzoate	Seeds	HPLC-UV	Tlili et al. (2015)
Trans-methyl cinnamate	Seeds, flower buds	HPLC-UV, GC-MS	Tlili et al. (2015), Romeo et al. (2007)
Benzoic acid	Flower buds, stems	LC-MS/NMR, HPLC-DAD	Mollica et al. (2019), Hu et al. (2017)
p-Hydroxybenzoic acid	Fruit, flower buds	HPLC-DAD	Yu et al. (2006), Li et al. (2014), Mollica et al. (2019)
Salicylic acid	Flower buds, stems	LC-MS/NMR	Mollica et al. (2019), Hu et al. (2017)
Methylparaben	Flower buds	GC-MS	Romeo et al. (2007)
vanillic acid	Fruit, flower buds	HPLC-DAD	Mollica et al. (2019), Yu et al. (2006)
Sinapic acid	Fruit	HPLC-UV	Aliyazicioglu et al. (2013)
Syringic acid	Flower buds	HPLC-DAD	Mollica et al. (2019)
Anisic aldehyde			
p-Coumaric acid			
o-Veratric acid			
o-Coumaric acid			
Trans-cinnamic acid			
Quinic acid	Leaves, aerial parts	LC-ESI-MS/MS	Bakr et al. (2016), Yahia et al. (2020)

## Terpenes and Miscellaneous

**FIGURE 6:**
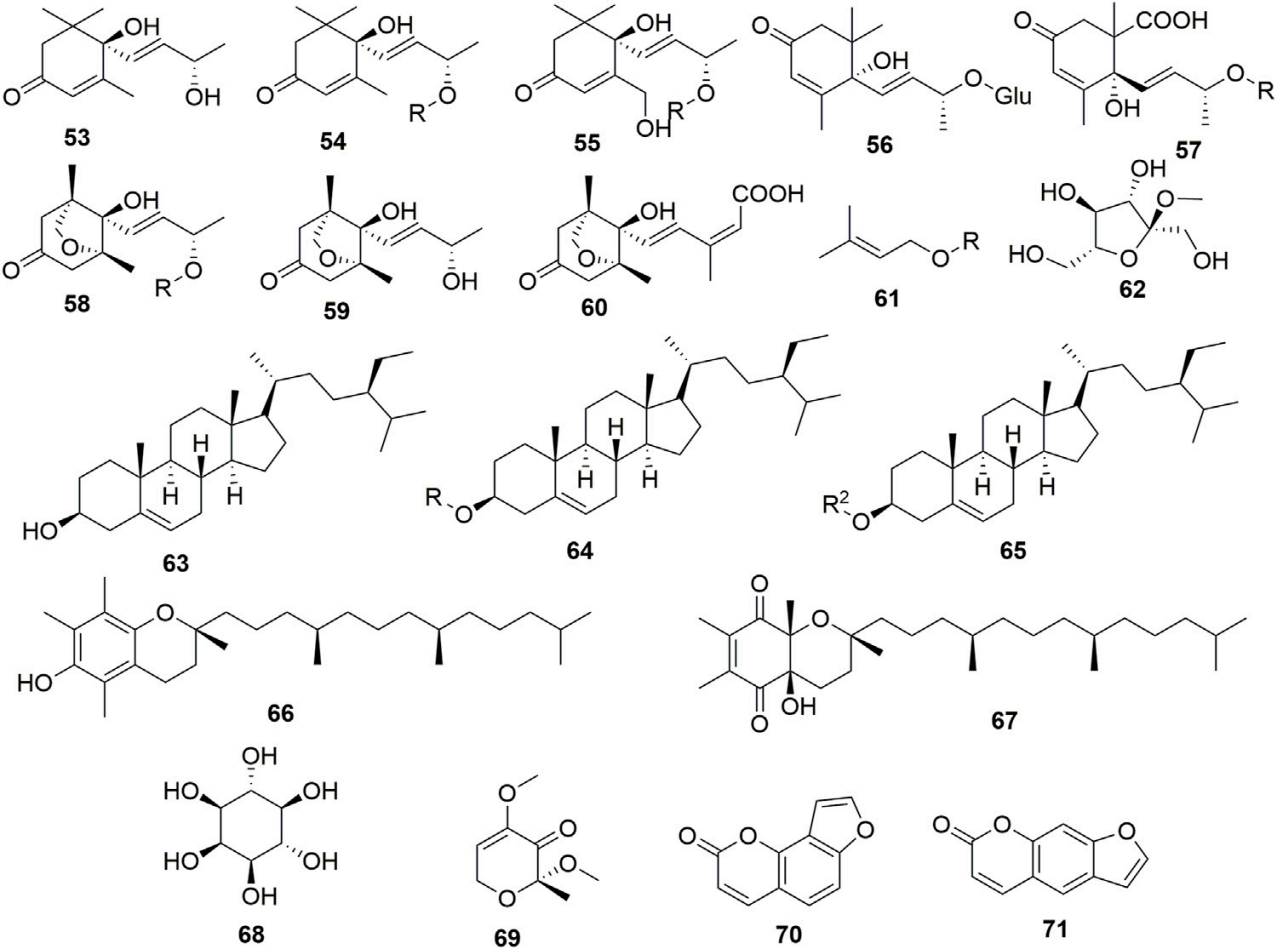
Terpenes and other compounds from Caper. R = *β*-D-glucopyranosyl, R^2^ = 6‘-stearyl-*β*-D-glucopyranosyl, **53** = (6*S*,7*E*)-6,9-Dihydroxymegastigma-4,7-dien-3-one, **54** = 4-Hydroxy-4-(3-hydroxy-1-buten-1-yl)-3,5,5-trimethyl-2-cyclohexen-1-one, **55** = (4*S*)-4-[(1*E*,3*S*)-3-(*ß*-D-Glucopyranosyloxy)-1-buten-1-yl]-4-hydroxy-3-(hydroxymeth…, **56** = Corchoionoside C, **57** = (2*R*)-2-[(1*E*,3*R*)-3-(*ß*-D-Glucopyranosyloxy)-1-buten-1-yl]-2-hydroxy-1,3-dimethyl-5…, **58** = (1*R*,5*R*,8*S*)-8-[(1*E*,3*S*)-3-(*ß*-D-Glucopyranosyloxy)-1-buten-1-yl]-8-hydroxy-1,5-dime…, **59** = (1*R*,5*R*,8*S*)-8-Hydroxy-8-[(1*E*,3*S*)-3-hydroxy-1-buten-1-yl]-1,5-dimethyl-6-oxabicycl…, **60** = Phaseic acid, **61** = Methyl *α*-D-fructofuranoside, **62** = 1-prenylglucopyranoside, **63** = *β*-Sitosterol, **64** = Daucosterol, **65** = Daucosterol 6'-*O*-stearate, **66** = alpha-tocopherol, **67** = (2*R*,4a*R*,8a*R*)-3,4,4a,8a-Tetrahydro-4a-hydroxy-2,6,7,8a-tetramethyl-2-(4,8,12-trimethyltridecyl)-2*H-*chromene-5,8-dione, **68** = myo-Inositol, **69** = (*R*)-2,4-Dimethoxy-2-methyl-6*H*-pyran-3-one, **70** = Isopsoralen, **71** = Sporalen.

C-15 sesquiterpene analogs (**53-60**) and steroids (**63-65**) (Figure 6) are two members of terpenes reported to have occured in Caper organs. The water-soluble fraction of the dried mature fruit material showed to contain the degraded tetraterpene analogs corchoionoside C, spionosides A and B, and phaseic acid along with the glucopyranosyl derivative (**61**) (Çalış et al., 2002). Moreover, Tlili et al. (2010) reported roughly 2.24 mg/g of phytosterols in the extracted lipids of the seeds plant. *β*-sitosterol was the most abundant in this series accounting for 1.39 mg/g of lipid followed by campesterol (0.382 mg/g) and stigmasterol (0.265 mg/g) (Tlili et al., 2010). However, only three sterols have been isolated by chromatographic methods including compound **63** and saponins (**64-65**). Two other sterol derivatives namely spinosols A and B have been highlighted to occur in Caper but there is no available information on their structures (Razaq et al., 2017).

Tlili et al. (2009) have also evaluated the wealth of the plant in carotenoids and tocopherols. The latter is present in plant seeds and constitutes 628 mg/100 g of the fatty oil extracted, made up of *γ*-tocopherol (92%), *α*-tocopherol (4%), and *δ*-tocopherol (2%) (Tlili et al., 2009). However, only α-tocopherol (**66**) and an oxidized analog (**67**) have been isolated to date but not *γ*-tocopherol although it is said to occur in higher amounts (Hu et al., 2017). In addition, the seed oil contained a significant level of β-carotene evaluated at 375 μg/100 g out of 457 μg/100 g of the plant carotenoids (Tlili et al., 2009). Caper also contains saccharides including sucrose, compounds **61** and **62**, pyran (**69**), polyol (**68**), and coumarins (**70-71**).

Nonetheless, leaves, seeds, roots, and fruits of Caper drain significant levels (0.02–48.7 mg/kg) of essential and nonessential heavy metals including iron (Fe), nickel (Ni), manganese (Mn), zinc (Zn), copper (Cu), cadmium (Cd), chromium (Cr), titanium (Ti), barium (Ba), strontium (Sr), aluminum (Al), magnesium (Mg), potassium (K), sodium (Na) and lead (Pb). These metals could originate from the *in-situ* bioremediation potency of the plant rather than its natural predisposition to produce them. However, the occurrence of a metal element in an organ varies from one research work to another. For instance, heavy metal content of Caper leaves found to decrease in the order of Fe > Zn > Mn > Cu > Pb > Ni > Cr > Cd by (Niaz et al., 2013) and of Cu > Ti > Cr > Ba > Zn > Sr > Mn > Al > Fe > Mg > K > Ca by (Vaidya et al., 2017), both in polluted and unpolluted aerials. The control of heavy metals in medicinal plants like Caper is essential to prevent their adverse effects on the human health system.

## Biological Potential of Caper

**FIGURE 7:**
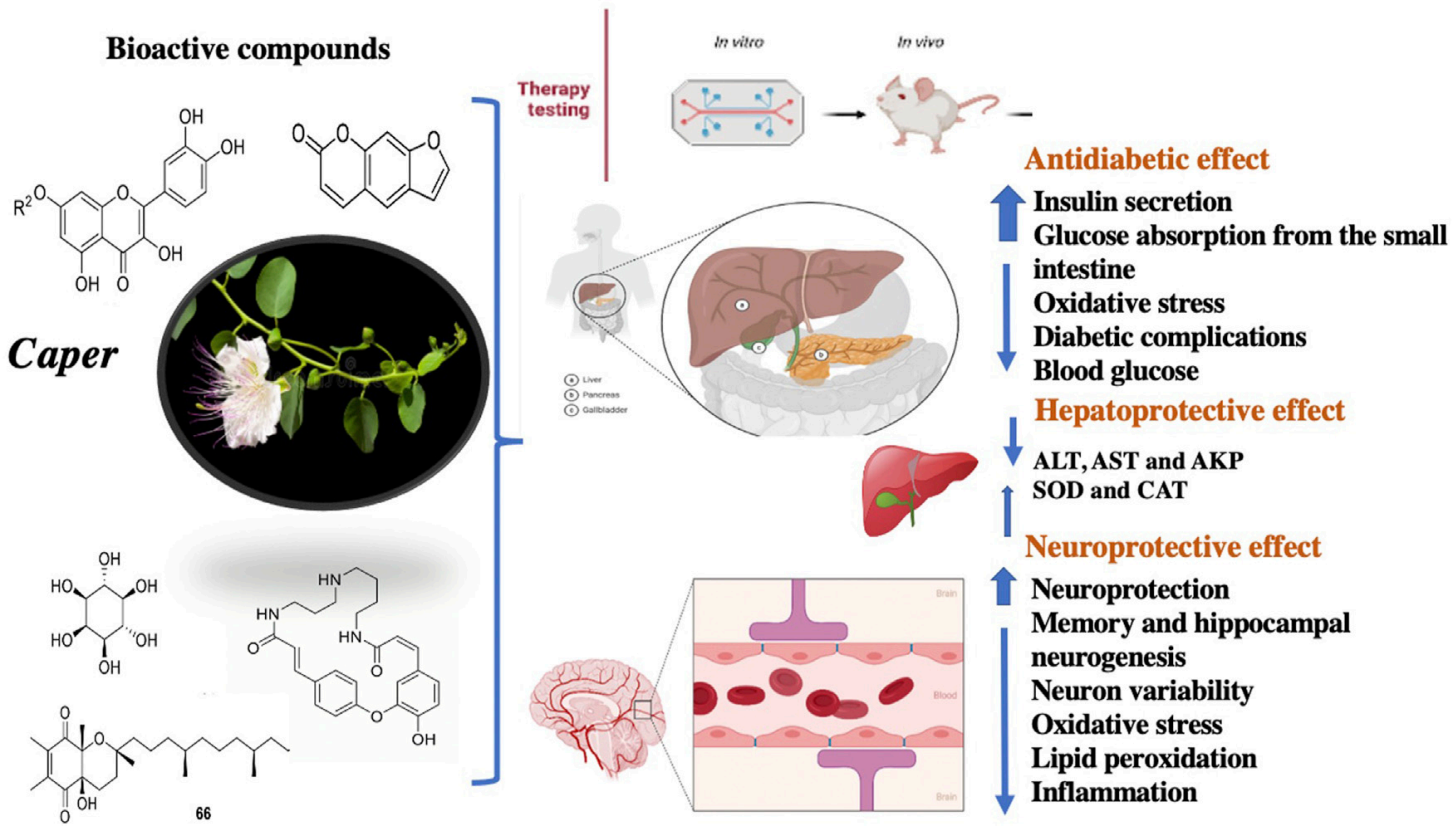
Selected biological activities of Caper.

Extracts and isolated compounds from various organs of Caper have been assessed for their biological potential. The activities were evaluated using common methodology and standards. Overall, Caper organs are preferably good antidiabetic, hepatoprotective, and neuroprotective agents (Figure 7). In addition, they also showed considerable antimicrobial, antioxidant, anti-inflammatory, and anticancer activities.

## Antimicrobial Activity

Caper extracts furnished promising antimicrobial activities in different experimental models (Supplementary Table S1). For instance, the methanol extract of the fruits showed a dose-dependent degree of quorum sensing, expressing 70–79% of biofilm inhibition and 46–67% reduction of exopolysaccharide production (EPS) production in *Serratia marcescens*, *Pseudomonas aeruginosa*, *Escherichia coli* and *Proteus mirabilis* at 0.5 and 2 mg/mL. The plant also reduced the swimming and swarming mobility of bacterial pathogens (Issac Abraham et al., 2011). Likewise, the antibacterial activity of the 80% hydroalcoholic (methanol, ethanol, and acetone) extracts from stem bark, shoots, fruits, flowers, and roots were investigated towards *Staphylococcus aureus* NCTC6571, *E. coli* ATCC8739, *Bacillus subtilis* NCTC10400, and *Pasteurella multocida* (isolated strains). The methanol extracts were the most significant to slow down the growth of the microorganisms with diameter zones of inhibition (DZI) around 24 mm compared to 27–32 mm for the controls, amoxicillin, and ciprofloxacin. The activity of the ethanol and acetone extracts was comparable with DZI of about 15 mm. None of the organs was most active than the others toward the tested microorganisms (Gull et al., 2015). Similar results were described by Adwan and Omar, (2021), stem and leaf ethanol extracts exhibited MIC of 6.25–100 mg/mL against relatively identical bacterial strains (Gull et al., 2015; Mickymaray and Al Aboody, 2019; Adwan and Omar, 2021). Similarly, the ethanol fruit extract of Caper featured strong antibacterial activity with MIC of 1.73 mg/mL against *Listeria monocytogenes* and 6.25 mg/mL against *E. coli* and *Pseudomonas aeruginosa*. The extract was moderately active against *S. aureus* sensitive to methicillin (SASM), *S. aureus* resistant to methicillin (SARM) *Klebsiella pneumoniae*, and *Salmonella* sp. with MIC 21–32 mg/mL. The ethanol flower extract was not as strong as the fruit extract against the same microorganisms disclosing MIC ranging from 10–15 mg/mL (Ennacerie et al., 2017; Hameed et al., 2021). Caper extract also showed differential antibacterial activity against *Pasteurella multocida*, *K. pneumonia, Acinetobacter baumannii, Enterobacter aerogenes,* and *Proteus mirabilis* with DZI values of 24.9, 36, 21, 26 and 27 mm, respectively (Gull et al., 2015; Mickymaray and Al Aboody, 2019; Adwan and Omar, 2021). From other perspectives, the copper nanoparticles prepared from the aqueous extract from the fruits showed significant antibacterial activity with MIC of 5–10 mg/mL against *S. aureus* PTCC1112, *B. cereus* PTCC1556, *E. coli* PTCC1330, and *K. pneumoniae* PTCC1053 (Ebrahimi et al., 2017). Likewise, silver nanoparticles prepared from aqueous fruit extract disclosed significant antifungal activity with MIC values ranging from 5 mg/mL to 0.625 mg/mL (Ebrahimi et al., 2019). Caper extracts rather were active against some fungal strains including *Aspergillus flavus*, *Candida albicans*, *Candida glabrata,* and *Kluyveromyces marxianus*. Overall, tested water-soluble or alcoholic extracts displayed moderate activity with either DZI around 19 mm or MIC <12.5 μg/mL (Sherif et al., 2013). All these promising antimicrobial results from Caper extracts are considered a good starting point for further studies to better understand the mode of action of these extracts (Supplementary Table S1).

## Antioxidant Potency

Antioxidants include all molecules able to inhibit free radical reactions produced naturally in the body from metabolic processes or by extrinsic agents such as exposure to X-rays, environmental pollution, ultraviolet light, drugs, and pesticides (Rahman, 2007). These free radicals cause damage to DNA, cell membranes, and other cell tissues leading to various diseases. Several studies revealed *in vitro* antioxidant potential of Caper extracts using DPPH, ABTS, FRAP, CUPRAC, phosphomolybdenum, metal chelating power, and TAC assays (Supplementary Table S2). These activities have been related to the occurrence of high levels of phenolic acids and flavonoids (Rahnavard and Razavi, 2017). Indeed, aqueous leaf extracts prepared by either maceration, reflux, or ultrasound-assisted extraction were assessed for their antioxidant activity. The reflux extract showed the highest capacity to reduce DPPH with IC_50_ of 36.6 mg/mL; the ultrasound-assisted extract showed the highest ABTS scavenging activity with 258.77 mg of ascorbic acid equivalent/g dry weight (DW), while the macerated extract showed the highest FRAP activity with EC_50_ equal to 120.2 mg/mL (Yahia et al., 2020). Likewise, the acetone 80% extract of fresh buds showed significant DPPH scavenging activity with IC_50_ = 5.90 μg/mL (El amri et al., 2019) as do the hydro-ethanolic extracts of different tissues towards DPPH radical with IC_50_ values of 1.41 mg/ml, 1.56 mg/ml, and 2.49 mg/mL, for leaves, fruits, and buds extracts, respectively (Assadi et al., 2021). In the same line, Saleem et al. (2021) compared the antioxidant activity of the methanol and dichloromethane (DCM) extracts of plant aerial parts and roots. The DCM extract showed the highest activity towards FRAP, CUPRAC, phosphomolybdenum, and metal chelating power assays with 50.37 mg TE/g extract, and 118.45 mg TE/g extract, 75.79 mg TE/g extract, and 2.51 mg EDTA/g endpoints, respectively. The methanol aerial parts and root extracts showed strong activity against DPPH and ABTS, instead. The administration of hydro-alcoholic extract of fruits demonstrated protective effects on tissue function through oxidative stress alleviation and antioxidant mechanism restoration (Mirzakhani et al., 2020).

## Hepatoprotective Activity

The liver plays an immense role in our body, it helps digest food, store energy, and get rid of poisons and toxins. Furthermore, the liver ensures the detoxification of a wide range of toxic molecules present in the organism. Most of these molecules increase the production of reactive oxygen species (ROS), which have exhibited their hepatotoxic effect in several experimental models (Sobeh et al., 2019; Sobeh et al., 2020a). Table 3 summarizes the hepatoprotective activity of Caper extracts.

**TABLE 3:** *In vivo* Hepatoprotective activity of Caper extracts.

Plant part	Extract	Dose	Inducer of liver damage	Effects	Ref.
Fruits	Aqueous	500 mg/kg	Paracetamol	The cytochrome P450 2E1% compared to control group: Paracetamol group = 249.28%	Ali Al-Nuani and Kadhim, (2020)
				Caper extract (CSE) = -18.72%	
				Paracetamol + CSE = 196.73%	
				The glutathione (GSH) % compared to control group: Paracetamol group = -53.80%	
				*Capparis* Spinosa extract = 0.61%	
				Paracetamol + CSE = -20.16%	
Leaves	Ethanolic (90%)	100, 200 and 400 mg/kg	CCl_4_	In comparison with the CCl_4_ receiving group, the extract significantly decreased AST and ALT levels at both concentrations of 200. and 400 mg/kg, while it increased the GSH levels only at 400 mg/kg	Kalantari et al. (2019)
				The extract significantly increased CAT activity and decreased MDA and ROS levels in comparison with the group treated with CCl4	
Leaves	Ethanolic (80%)	100, 200 and 400 mg/kg	*Tert*-butyl hydroperoxide (T-BHP)	At all doses, Caper extract decreased MDA levels and alleviate GSH diminution caused by T-BHP.	Kalantari et al. (2018)
				All doses increased SOD and CAT activities compared to the mice treated with T-BHP (no specific data)	
Leaves and Fruits	Methanolic	200 and 400 mg/kg	CCl_4_ (30% mixed with olive oil. 1 mL/kg)	No significant difference in body and liver weight was noticed comparing the group treated with CCl_4_ with the group treated with the extract	Aichour et al. (2018)
				The leaves and fruits extract decreased AST, ALT, and ALP levels in comparison with the groups treated with CCl4. The best results were observed at 400 mg/kg in both extracts	
Leaves	Methanolic	500, 1000, and 2000 mg/kg	No inducer was used	The methanolic extract of leaves showed no significant differences in liver safety biomarkers including alkaline phosphatase (ALP). aspartate aminotransferase (AST) and alanine aminotransferase (ALT)	Eltawaty et al. (2018)
Leaves	Methanolic	200 mg/kg	CCl_4_	AST level	Tlili et al. (2017)
				Control = 121.3 U/L	
				ccl_4_ = 141.8 U/L	
				ccl_4_ + MECS = 136.5 U/L	
				MECS = 124.2 U/L	
				ALT level	
				Control = 81.38 U/L	
				ccl_4_ = 112.8 U/L	
				ccl_4_ + MECS = 93.98 U/L	
				MECS = 78.76 U/L	
				LDH level	
				Control = 952.6 U/L	
				ccl_4_ = 1129 U/L	
				MECS = 947.9 U/L	
				ccl_4_ + MECS = 1032 U/L	
Aerial Part	Ethanolic 70%	200 and 400 mg/kg	Thioacetamide (TAA)	The level of liver MDA significantly declined while the GSH activity was significantly elevated after treatment with the extract at 200 and 400 mg/kg while the group treated with TAA showed higher levels of MDA and lower levels of GSH.	Yusufoglu et al. (2014)
				The extract induced the augmentation for 3 weeks of ALT. AST. ALP. γ-GT and BRN levels decreased by TAA treatment	
				Activities of SOD. CAT. and GPx enzymes were significantly increased following treatment of rats with the extract at both concentrations of 200 and 400 mg/kg	
				At all treatments, the dose of 400 mg/kg showed the best results	

MECS, methanol extract of Caper.

The methanol leaf and fruit extracts of Caper were reported to display a significant hepatoprotective effect that may lead to the stoppage of the extension of liver damage by increasing levels of phase I detoxification enzymes namely cytochrome P450 enzymes (CYP) and phase II detoxification enzymes such as glutathione S-transferase (GST), quinone reductase (QR), UDP-glucuronosyltransferase (UGT), amino acid transferases, N-acetyl transferases, and methyltransferases. In addition, Caper extracts were able to decrease other enzyme levels that the liver releases in response to damage or disease including ALT, AST, AKP, γ-glutamyltransferase (γ-GT), and lactate dehydrogenase (LDH). Aichour et al. (2018), for instance, pointed out that methanol extracts from leaves and fruits of Caper reduced the elevated serum enzyme (AST, ALT, ALP, and bilirubin) levels induced by CCl_4_ at doses of 200 and 400 mg/kg for the highest effect encountered at 400 mg/kg (Aichour et al., 2018). Similarly, the hepatoprotective activity of the ethanol leaf extract of Caper was evaluated against tert-butyl hydroperoxide (T-BHP) as a liver damage inducer. The extract mitigated the diminution of detoxification enzyme GSH and reduced MDA levels while SOD and CAT levels increased significantly in the group treated with the extract (Kalantari et al., 2018).

The protective effects of Caper seed extract on the toxicity induced by CCl_4_ and cisplatin were demonstrated by Tir et al. (2019). Mainly, the pretreatment of animals restored the biomarkers of liver and kidney injuries alongside an increase in antioxidant enzymes, which was confirmed by the histopathological studies with a decrease in the degree of tissue fibrosis (Tir et al., 2019). Recently, Ali Al-Nuani and Kadhim, (2020) investigated the effect of aqueous fruit extract of Caper on two detoxification enzymes in comparison with the paracetamol effect. The extract furnished a reduction of the cytochrome P450 2E1 percentage from 249.28 to 196.73% when paracetamol is injected first then the extract and from 249.28 to 200.59% when the extract is injected prior to paracetamol. The glutathione (GSH) levels also increased to 53.80% with paracetamol and 20.16 and 40.49% when paracetamol was injected followed by extract and when the extract was injected first followed by paracetamol, respectively (Ali Al-Nuani and Kadhim, 2020). Nevertheless, further studies on the mechanism of action of Caper in liver diseases are required to shed light on developing methods in clinical practices.

## Anti-Inflammatory Activity

Inflammation occurs in the body as a natural defense mechanism against xenobiotics and harmful compounds, but it can also cause diseases (Sobeh et al., 2020b). Various studies reported the anti-inflammatory effect of Caper extract including *in vivo* and *in vitro* assays. Kernouf et al. (2018) revealed the anti-inflammatory activity of the 80% hydromethanolic extract of buds at 200 and 400 mg/kg doses. The extract reduced the *in vitro* paw edema inflammation by 52–69% compared to the control, while 1 mg/pouch of the extract revealed inhibition of 48.92% of leukocytes infiltration. In addition, 100 μg/ml of the extract mitigated the production of inflammatory mediators TNF-α, IL-1β, LTB4, and superoxide anion by 21.28, 38.04, 20.84, and 71.16%, respectively (Kernouf et al., 2018). Likewise, the methanol extract and the resulted hexane fraction of the plant leaves comparably reduced contact hypersensitivity response in mice by approximately 73.44% of edema inhibition percentage using 1.07 g/kg dose and inhibited IFNγ, IL-17, and IL-4 as cytokine gene expression (El Azhary et al., 2017).

## Anticancer Activity

Cancer is an abnormal growth and proliferation of cells and affects any human organ. Lung, prostate, colorectal, stomach, and liver are the most common organs likely to develop cancer in men, while breast, colorectal, lung, cervical, and thyroid cancers are the most common types of tumors found among women. Natural products and related constituents could serve as promising alternatives to chemotherapeutic and chemopreventive agents (El-Hossary et al., 2020; Tawfeek et al., 2021). A study has been conducted earlier by Yu et al. (2010) against human gastric cancer cell SGC-790 with MTT assay, the n-butanol extract showed potential effect against cell proliferation, the lowest and the highest inhibitory rate percentages were 24.1% at 5 μg/mL and 75.4% at 400 μg/mL, respectively, with an IC_50_ of 31.5 μg/mL (Yu et al., 2010). Saleem et al. (2021) investigated the cytotoxicity of the methanol and dichloromethane extracts of aerial parts and roots against breast cancer cell lines MDA-MB 231 and MCF-7. The dichloromethane extract of the roots showed the highest activity against MB 231 cells with a viability percentage of 73.81%, while the dichloromethane extract of the aerial part was active against both cells with a viability yields of 55.36 and 55.72% against MB 231 and MCF-7, respectively (Saleem et al., 2021). Moreover, Caper isothiocyanates are well known as cancer preventive agents and different extracts have hypoglycemic properties and protective effects against hepatotoxic substances. These promising *in vitro* results noted by different extracts (Table 4) need further *in vivo* studies to prove their efficiency and uncover the underpinning mechanisms of antitumor activity.

**TABLE 4:** *In vitro* anticancer effects of *Caper* extracts.

Plant part	Extract	Cell lines	Effects	Reference
Aerial parts	Methanolic	Breast cancer cell line: MDA-MB 231 and MCF-7	MB 231 viability: 47.84%	Saleem et al. (2021)
			MCF-7 viability:12.59%	
	Dichloromethane extract		MB 231 viability: 55.36%	
			MCF-7 viability: 55.72%	
Root parts	Dichloromethane extract		MB 231 viability: 73.81%	
			MCF-7 viability: 48.46%
	Methanolic extract		MB 231 viability: 46.98%
			MCF-7 viability:2.67%
Aerial parts	Ethanolic (80%)	Cancer cells: Hela. MCF7 and Saos	The most effective dose of the drug for cancer cells compared to normal cells was 250 μg/ml after 72 h	Moghadamnia et al. (2010)
ND	*N*-butanol	Human Gastric Cancer cell: SGC-7901	IC_50_ = 31.542 μg/mL Inhibitory rate % = 24.105; 15.297; 63.759; 67.661; 70.948; 75.424 respectively for the concentration 5; 25; 50; 100; 200 and 400 μg/mL	Yu et al. (2010)

ND, Not determined. All experiments were done using MTT assay.

## Neuroprotective Effect

Studies have highlighted the relationship between inflammation and memory impairments (Geng et al., 2018; Lin et al., 2018). Systemic inflammation results in learning and memory impairment through the activation of microglia. Caper reduced brain inflammation by an increase in anti-inflammatory mediators (IL-10) and a decrease of inflammatory mediators (TNF-α, IL-1β) in LPS-induced cognitive impairment (Rahimi et al., 2020). In the same line, Goel et al. (2016) investigated the effect of Caper on learning and memory damage after administration of LPS. Results demonstrated a reductive effect of the aqueous extract of the plant buds on the neurodegeneration in the hippocampal circuit region of the hippocampus (Goel et al., 2016). The inhibition capacity of Caper (200 mg/kg b.w.) attenuated cognitive impairment induced by D-galactose in mice (Turgut et al., 2015). Furthermore, Khorrami et al. (2021) investigated the effect of Caper extract in the middle cerebral artery occlusion (MCAO) model of ischemic stroke. As a result, the pretreatment with Caper reduced MVAO injury and the neurological deficit score through the suppression of oxidative stress (Khorrami et al., 2021). Moreover, Caper extract demonstrated a good effect to regulate inflammation-involved genes in Alzheimer’s, especially on the amyloid-beta peptide (Aβ)-injected rats. This activity could be attributed to the high level of flavonoids in the plant (Mohebali et al., 2018). The findings demonstrated the neuroprotective effect of Caper and provide evidence that the plant could be considered for the treatment of neurodegenerative disorders such as Alzheimer’s disease.

## Antidiabetic Activity

The antidiabetic properties of Caper extracts have been well-documented in the literature and are summarized in Table 5. Studies were carried out *in vivo* using animal models and clinical trials in patients and demonstrated the antihyperglycemic effects of Caper at various doses starting from 15 mg/kg up to 800 mg/kg and between 12 and 60 days of treatment (Rahmani et al., 2013; Kazemian et al., 2015; Eddouks et al., 2017).

**TABLE 5:** *In vivo* antidiabetic effects of *Caper* extracts.

Plant part	Extract*	Doses	Effect	Reference
Stz-Induced Diabetic Rats				
Fruits	Ethanolic extract 70%	200 and 400 mg/kg	Extract treatment at doses of 200 and 400 mg/kg produced a significant reduction of fast blood glucose levels by 16 and 20% respectively compared to diabetes control. Significant effect on lipid profile observed only at 400 mg/kg	Assadi et al. (2021)
			Alleviate liver oxidative stress by increasing significatively CAT. GSH-Px and GR activities in both concentrations and a significant increase in GST activity only at 400 mg/kg	
Leaves, Fresh Buds, and Salty Buds Oral	Methanolic (60%)	100,200 and 400 mg/kg	Leaf and bud lowered blood glucose level	Mollica et al. (2017)
			at 100,200 and 400 mg/kg	
			Significant decrease in biochemical parameters of the liver (ALT and AST) and kidney (urea and creatinine) in rat groups treated with Caper leaf and bud at all concentrations	
			Decrease in Total cholesterol and Triglyceride levels in rat groups treated with Caper leaf and bud at all concentrations	
Fruits	Aqueous	20 mg/kg	Decrease blood glucose level dropped from 19.81 to 10.57 and 5.59 mM two and 6 h after a single oral administration and dropped by 39% from 19.81 to 11.96 mM	Eddouks et al. (2017)
			No significant differences in basal metabolic clearance rate (MCR) of glucose between the CS-treated diabetic group and the control group	
Roots	Ethanolic (70%)	200 and 400 mg/kg	The decrease in blood glucose levels in both concentrations started from the third week until the end of the study	Kazemian et al. (2015)
			Cholesterol levels decreased in both concentrations compared to diabetic control	
			Reduction of liver enzymes activity was observed in both concentrations in the case of ALT and ALP. and only in 0.2 in AST activity	
Fruits	Ethanolic (70%)	200 and 800 mg/kg	Both concentrations managed to reduce blood glucose levels compared to the control group. the reduction was dose-dependent	Rahmani et al. (2013)
			Significant reduction in triglycerides level at both concentrations. and a significant reduction of cholesterol level was observed only at 200 mg/kg	
Alloxan-Induced Diabetic Rats				
Leaves	Ethanolic (80%)	200,400,800 mg/kg	Significant blood glucose level was registered after 8 h only in 400 and 800 mg/kg doses	Hussain et al. (2017)
			The repeated oral dose (400 mg/kg) of CS leave extract significantly reduced the blood glucose level at the 1st. 2nd and 4th week of treatment	
Leaves	Polyphenolic	15 and 25 mg/kg for 28 days	Decrease in fasting blood glucose	Oudah et al. (2014)
Fruits	Hydroalcoholic	300 mg/kg for 12 days	Decrease in fasting blood glucose and damage in the pancreas and liver	Hashemnia et al. (2012)

*All extracts were given orally.

The hydroethanolic fruit extract of Caper produced a significant reduction of fasting blood glucose levels in type-2 diabetic rats by 16% at a concentration of 200 mg/kg and by 20% at 400 mg/kg compared to streptozotocin (STZ). However, a significant effect on lipid profile was observed only at the concentration of 400 mg/kg, mitigating liver oxidative stress and increasing detoxification enzyme levels (Assadi et al., 2021). Similar results were found earlier by (Kazemian et al., 2015) using 70% ethanolic extract of the roots of Caper at the same doses (200 and 400 mg/kg). In the same line, Jalali et al. investigated the antidiabetic effects of aqueous extract of the fruits and revealed the oral administration of 20 mg/kg of the extract decreased the fasting blood glucose (FBG) in STZ-induced diabetic rats (Jalali et al., 2016).

Another study showed a better hypoglycemic effect using a lower dose of aqueous extract (20 mg/kg) with a decrease in blood glucose level from 19.81 to 5.59 mM after a single oral administration and to 11.96 mM after a daily repeated administration (Eddouks et al., 2017). A mixture of plant materials including Caper was used to assess their efficacy on patients with Type-2 diabetes mellitus, the plant mixture reduced fasting plasma glucose and glycated hemoglobin (HbA1c) compared to the patients treated with placebo and showed similar results compared to the metformin-treated patients (Mehrzadi et al., 2020). In addition, Huseini et al. (2013), evaluated the antihyperglycemic effect of Caper in patients with type-2 diabetes and the results showed a significant decrease in FBG and HbA1c levels in the patients treated with 400 mg of hydroalcoholic extract of the plant. Based on these findings, Caper could be considered an adjuvant agent in diabetes.

## Biological Activities of the Phytocompounds Isolated From Caper

Different parts of Caper comprise a wide variety of active secondary metabolites with several biological activities. Many bioactive compounds were isolated from Caper. Although phytochemicals act synergistically with other compounds in the plant, some of the identified compounds demonstrated a variety of biological activities. Table 6 shows a summary of bioactive compounds isolated from Caper.

**TABLE 6:** Biological activities of the phytocompounds isolated from Caper.

Biological activities	Compounds	Bioassay	Results	References
Hepatoprotective activity	p-Methoxy benzoic acid	CCl_4_ and Pcl induced hepatotoxicities	Treatment with 30 mg/kg (b.w.) resulted in 89.68, 105.28, 78.91, 56.55 and 137.4, 86.30, 92.91, 62.55% reductions in SGPT, SGOT, Alkp and T. Bil levels	Gadgoli and mishra (1999)
Antioxidant activity	Cappariside	DPPH assay	IC_50_ = 0.204 ± 0.002 mM	Yang et al. (2010b)
	*E*-Butenedioic acid		IC_50_ > 1 mM	
	Ethyl 3,4-dihydroxybenzoate		IC_50_ = 0.011 ± 0.0 mM	
	5-Hydroxymethylfurfural		IC_50_ > 1 mM	
	5-Hydroxymethyl furoic acid		IC_50_ > 1 mM	
Allelopathic activity of *Lactuca sativa*	Quercetin 3-*O*-β-D glucopyranoside	Germination Index (%)	GI = 76.4 ± 9.1	Ladhari et al. (2013a)
			RL = 30.4 ± 0.9	
			SL = 37.4 ± 2.4	
	Kaempferol 3-*O*-β-D-glucopyranoside	Root length (%)	GL = 90.9 ± 2.6	
			RL = 51.4 ± 3.7	
			SL = 5.7 ± 3.1	
	Quercetin	Shoot length (%)	GL = 4.8 ± 3.6	
			SL = 54.1 ± 3.7	
Anti-inflammatory activity	Ginkgetin	NF-kB activation	IC_50_ = 7.5 ΜmM	Zhou et al. (2011)
Anti-arthritic activity	Stachydrine	Nociception induced by acetic acid and hot-plate, and inflammation induced by carrageenan and xylene	Delay the response to thermal stimulation	Feng et al. (2011)
			Inhibited the abdominal constriction response caused by acetic acid	
			Reduced ear and paw edema caused by xylene and carrageenan	
Nematocidal activity	2-Thiophenecarboxyaldehyde	Induce paralysis in second-stage nematode juveniles (J2)	EC_50_ = 7.9 mg/L	Caboni et al. (2012)
			EC_50_ =14.1 mg/L	
	Methyl isothiocyanate			
Antimicrobial activity	1-methyl-2-butyl-pyrrolidine	*E. coli*	The compounds demonstrated a very good activity for inhibition of pathogenic bacteria (*E. coli* and *S. aureus*)	Abd Al-Majeed et al. (2016)
	2- pyrrolidineethanamine			
	2,3 dihydroxy-6-methyl-4H-pyrane-one, 5-oxo-pyrorrolidine, 2-(2-hydroxyethyl) piperidine			
	Aziridine			
	Piperidine-4-ol			
	3-Piperidinol	*S. aureus*	Very good inhibition zone diameters were detected	
	2-Methyl aziridine			
	1-(2-Butenyl) pyrrolidine			
	2*H*-1-Benzopyran-2-one			
	2,4-Dimethoxy-5-pyrimidine carboxyaldehyde			
Antimicrobial activity	(7,11,15,19)—Ethyl 4,8,12,16,20- pentamethyldocosa -7,11,15,19—tetraenoate	*S. Aureus*	MIC = 100 mg/mL	Arean et al. (2019)
		*S. epidermidis*	MIC = 100 mg/mL	
		*K. pneumoniae*	MIC = 50 mg/mL	
		*E. coli*	MIC = 100 mg/mL	
		*Salmonella*	MIC = 100 mg/mL	
	Methyl 2′,15′-dimethyl- 5,5′- dioxo- 18′- oxaspiro[oxolane-2,14′-pentacyclo-octadecan]-7′-ene-9′- carboxylate	*S. Aureus*	MIC = 200 mg/mL	
		*S. epidermidis*	MIC = 200 mg/mL	
		*K. pneumoniae*	MIC = 200 mg/mL	
		*E. coli*	-	
		*Salmonella*	-	

## Toxicity Studies

Only a few reports described the side effects of Caper (Taghavi et al., 2014; Kazemian et al., 2015). All in all, Caper is safe for consumption. Fruits induce no side effects on the liver and no signs of nephrotoxicity in rats (Heidari et al., 2010; Huseini et al., 2013). Ouadah Hamam et.al. (2019) investigated the acute toxicity effect of polyphenolic extract of the plant leaves and the extract was nontoxic at doses up to 100 mg/kg b.w (Oudah hamam et al., 2019). The hydroalcoholic extract of Caper fruits in rats showed an LD_50_ value of 400 mg/kg supporting results encountered by El-Hawary et al. (2018) who demonstrated no mortality in rats within 24 h of pretreatment with Caper methanol extract at doses of 1000–4000 mg/kg, suggesting an LD_50_ > 4000 mg/kg (El-Hawary et al., 2018).

## Other Biological Activities

Caper extracts were also screened for other biological activities including insecticidal effects. Caper roots are submerged in water all night and then dispersed on plant seeds to protect the seeds from pests all over the year (Mahboubi and Mahboubi, 2014). In addition, Caper leaf extracts displayed strong insecticidal activity with 100% mortality, while stem extracts showed moderate activity (50% of mortality) (Ladhari et al., 2013b). The acetone extract of Caper showed insecticidal activity against third instar larvae of *Aedes aegypti* inducing 40% mortality at 2 mg/ml concentration and an LC_50_ value of 1.77 mg/ml. In addition, the hydro-alcoholic extract of Caper showed significant *in vivo* hypnotic activity in mice in comparison with diazepam at a 3 mg/kg body weight dose inducing no cytotoxic effect and an LD_50_ value of 2.4 g/kg (Rakhshandeh et al., 2020).

## Clinical Studies

Various studies conducted on Caper showed high promising results, which led to clinical studies to confirm previous *in vivo* and *in vitro* results. A study by Banerjee et al. (2011) carried out using Caper as a part of the polyherbal formulation to assess its efficiency and safety profile as an antioxidant for geriatric patients. A remarkable restoration of antioxidant properties was reported among the patients treated with the polyherbal formulation in comparison with the control group (Banerjee et al., 2011). Another clinical trial investigated the anti-hyperglycemic effects of the Caper fruit extract in type 2 diabetic patients and significant results were seen in patients treated with 400 mg extract with no side effects on the kidney or liver (Huseini et al., 2013).

## Commercial Formulation and Patented Products of Caper

Caper is one of the most important economical species in the Capparaceae family. Caper and its berries are the main products with economic importance at an international level. They are used as a flavor in food industries or as condiments (Rivera et al., 2002; Reyahi-Khoram and Reyahi-Khoram, 2018). In countries such as Tunisia, Saudi Arabia, Lebanon, and Syria, the species is suggested to raise the socioeconomic value (Sher and Alyemeni, 2010). In China, Caper constituted an annual contribution to the economy of the country of about 3 million USD. In the Balkans region, the total production costs of Caper represent less than 10% only of its selling price in the US markets (Saadaoui et al., 2011; Saadaoui et al., 2013). In addition, the plant has high nutritional value and demonstrated efficacy in medicines and cosmetics manufacturing (Aytac et al., 2009). For instance, Gatuline® Derma-Sensitive is a natural soothing active prepared from Caper fruit extract. The product is an anti-aging agent claiming skin protection and reducing inflammation. Moreover, the product is commercialized under SKIN MOON®; SKIN SAVE® and certified by ECOCERT and NSF. Caper buds are also typically commercialized as delicatessen products and used as pickles. The buds are categorized consistent with their sizes, the smallest is the most expensive in the market due to their concentrated flavor (Peregrin, 1985). Moreover, the young shoots of Caper are cooked in the same way as asparagus (Facciola, 1990).

## Bioavailability and Pharmacokinetics of Caper Phytoconstituents

**FIGURE 8:**
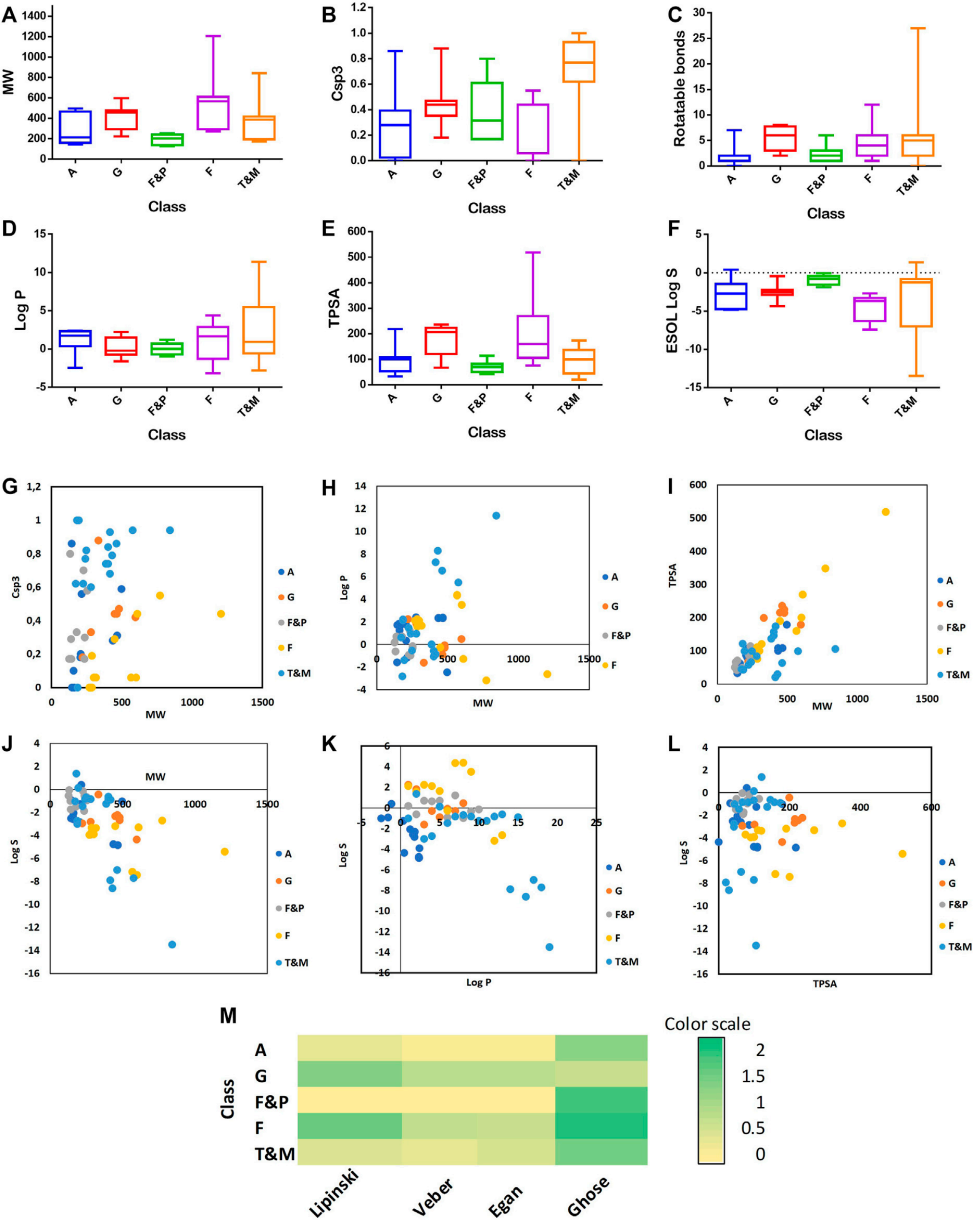
Distribution of MW **(A)**; F Csp3 **(B)**; number of RBs **(C)**; Log P **(D)**; TPSA **(E)**; and Log S **(F)** accordingly to the class of compounds. Comparison between the values of F Csp3 carbons and MW **(G)**; log P and MW **(H)**; MW and TPSA **(I)**; MW and log S **(J)**; log P and log S **(K)**; TPSA and log S (**L**). Heatmap of the compliance with rules of drug-likeness for the compound’s classes **(M)**. A (Alkaloids), G (Glucosinolates), F&P (Furans), F (Flavonoids), and T&M (terpenoids and miscellaneous).

Caper furnished numerous biological activities, among them antioxidant, antibacterial, antifungal, anti-inflammatory, analgesic, antitumor, hepatoprotective, and neuroprotective effects. These activities are attributed to the presence of diverse classes of secondary metabolites, such as phenolic acids, glucosinolates, furans, pyrroles, flavonoids, alkaloids, and terpenoids. To draw insight into the possible mechanisms, several molecular descriptors were calculated using SwissADME (Daina et al., 2017), among them several drug-likeness rules (Lipinski, Veber, Egan, and Muegge). Interestingly, out of 16 alkaloids identified from the plant, 14 compounds fulfilled all criteria of Lipinski, Egan, and Veber rules, and 7 and 3 compounds fulfilled Muegge and Ghose rules, Supplementary Table S3 and Figure 8. Also, all furans and pyrroles (10 compounds) satisfied all criteria of Lipinski, Egan, and Veber rules, and 5 and 2 compounds satisfied Muegge and Ghose rules. Most of the terpenoids and the other miscellaneous compounds fulfilled the criteria of the five tested drug-likeness rules as well. However, most of the flavonoids did not obey the rules due to their high molecular weights, and high numbers of hydrogen bond donors and acceptors. Glucosinolates showed some violations of the tested rules and this was partially due to their high numbers of hydrogen bond donors and acceptors, Supplementary Table S3 and Figure 8.

Topological polar surface area (TPSA), another descriptor, is the sum of the surfaces of all the polar atoms present in a molecule, which mainly are the oxygen and nitrogen atoms including the attached hydrogens. Apart from the molecular weight, TPSA has a great impact on the ability of a molecule to penetrate through the cell membranes and blood-brain barrier. Veber states that molecules with TPSA ≤140 A^2^ tend to be well absorbed and able to reach their molecular target within the body cells. Veber also stated that a molecule should have no more than 10 rotatable bonds for good oral absorption. Egan considered chemical compounds with TPSA not more than 132 A^2^ and log-P between -1 and 6 as leads with high drug-likeness potential and good oral bioavailability. Muegge utilized a pharmacophore point filter based on very simple structural rules to differentiate between drug-like and nondrug-like molecules, among them TPSA not greater than 150 A^2^ as well as no more than 15 rotatable bonds. Noteworthy, all furans and pyrroles, terpenoids and miscellaneous compounds and alkaloids, except compounds 9 and 21, had TPSA ≤150 A^2^. All Caper compounds, except one terpenoid (compound 65) had rotatable bonds less than 15, Supplementary Table S4 and Figure 8.

Another indicator for oral bioavailability that we tested is the bioavailability score which indicates the possibility of a molecule to be more than 10% bioavailable in the absorption assays. Generally, compounds satisfying the Lipinski rule with a bioavailability score of 0.55 are considered to be orally bioavailable. Out of the identified compounds from the plant, 50 phytoconstituents showed a bioavailability score of 0.55. Compounds 11, 13, 14, and 32, are of special interest as they showed good bioavailability scores of 0.56, 0.85, 0.56, and 0.85, respectively, Supplementary Table S4 and Figure 8.

Oral bioavailability depends as well on the degree of the molecular flexibility of a given compound. Compounds with very a high degree of flexibility do not usually show good oral bioavailability as they tend to be less planar and with very complex 3D shapes. The sp^3^ carbons fraction (Fraction Csp^3^) and the number of rotatable bonds (RB) are two crucial measures for molecular flexibility. Csp^3^ is the ratio of the sp^3^ carbon atoms to the total carbons present in a given compound. It assigns the degree of carbon saturation, characterizes the space complexity, and also correlates to the solubility of the compound. A Csp^3^ score between 0.25 and 1 is considered optimum for drug likeness. In the studied case, 48 phytoconstituents showed a Csp3 score ranging between 0.28 and 1. The water solubility, expressed as log S, is another essential measure for drug bioavailability. Compounds with poor water solubility have poor absorption and oral bioavailability, and low formulation potential. Caper phytoconstituents furnished different solubility orders as furans and pyrroles, were the most soluble class (mean value of −0.8), while flavonoids were the most poorly soluble class (mean value of −3.7), Supplementary Table S4 and Figure 8.

To elucidate the pharmacokinetic behavior of the identified secondary metabolites from Caper, various descriptors were explored. These include gastrointestinal absorption (GI), blood-brain barrier permeation (BBB), P-glycoprotein substrate (P-gp), skin permeation (Log Kp), and potential inhibitors of cytochrome P450 members, Supplementary Table S5 and Supplementary Figure S1. Interestingly 14 compounds displayed high GI absorption, passively crossed the blood membrane barrier, and did not show any potential for P-glycoprotein substrate, Supplementary Table S5, and Supplementary Figure S1. Also, Caper constituents revealed potential inhibition for some CYP 450 isoforms which require attention when coadministered with possible substrates of these enzymes, Supplementary Table S5.

Altogether, Caper is rich in phytoconstituents that fulfilled all the criteria of several drug-likeness rules with promising pharmacokinetic behavior which promotes its utilization as well as further research to isolate its phytoconstituents and evaluate their biological activities.

## Discussion

This review aimed to summarize the scientific literature on the nonconventional edible Caper plant (*C. spinosa*) and evaluate the phytochemistry, safety, and biological activities of its extracts and/or phytocompounds. The major phytochemicals identified in Caper were flavonoids (rutin, quercetin, and catechin), alkaloids (indoles and spermidines), and glucosinolates (glucocapparin). Other constituents such as furan and pyrrole derivatives as well as polyunsaturated fatty acids represented mainly by oleic acid, linoleic acid, and palmitic acid were also among the most important group of chemicals found in Caper. This plant is well renowned for its ethnopharmacological interests mainly in Iran, China, and India but also in Morocco with very diversified applications ranging from rheumatism and correlated infections to diabetes and kidney stones (Table 1). Several studies supported these traditional uses *via in vitro* and *in vivo* studies. Given its biosafety both traditionally and through scientific studies, Caper’s different extracts have been shown to elicit strong antioxidant activities *in vitro* and related disorders due to the occurrence of high levels of phenolic acids and flavonoids. Compounds isolated from Caper such as 5-hydroxymethylfurfural, *E*-butenedioic acid, and 5-hydroxymethyl furoic acid were demonstrated as DPPH scavengers (Table 6). This could be behind the protective effect of Caper extracts in alleviating the dysregulation of hepatic enzymatic parameters and in boosting the antioxidant machinery following stress induction. Neuroprotective effects were also demonstrated using Caper aqueous extract which attenuated the cognitive impairment and reduced the middle cerebral artery occlusion (Khorrami et al., 2021). This was noticed in the inflammation process involving Alzheimer’s genes as well. However, more in deep studies should address the underpinning mechanisms and uncover the targets of Caper antioxidant compounds within the cells and explore their downstream effects.

The most well-documented effect of Caper is the antidiabetic activity. It was shown *in vivo* using animal models and clinical trials in patients. A decrease in FBG and HbA1c were the main induced effects (Huseini et al., 2013). These encouraging results strongly support the use of Caper extracts as adjuvant agents in diabetes treatments. Caper also furnished anti-inflammatory responses both *in vitro* and *in vivo* as it inhibited the edema inflammation, reduced leukocyte infiltration, mitigated the production of pro-inflammatory mediators (TNF-α, IL-1β, and LTB4), and increased anti-inflammatory mediators (IL-10) (Kernouf et al., 2018). It has been shown that Nf-KB activation by ginkgetin, a compound isolated from Caper, is an interesting mechanism of anti-inflammatory responses (Zhou et al., 2011). However, more studies on other Caper extracts and/or essential oils on inflammation using cell-based lines and animal models are necessary.

Additionally, an exhaustive number of *in vitro* studies have shown that Caper extracts had anticancer and antimicrobial properties and compounds like 1-methyl-2-butyl-pyrrolidine, 2-methyl aziridine, aziridine, 7,11,15,19−ethyl 4,8,12,16,20-pentamethyldocosa-7,11,15,19−tetraenoate were identified as the bioactive agents (Table 6). As suggested, these plant extracts/compounds could trigger the bacterial cell membrane, coagulate the cytoplasm and bind lipids and proteins (Viuda-Martos et al., 2011). The cytotoxic effect of phytochemicals has been reported to be a result of many mechanisms including the activation of the apoptosis-inducing enzymes (Caspases 3, 8, and 9) of cancer cell lines and the expression of death receptors (Khan et al., 2020; Guesmi et al., 2021). However, given the complexity of biological systems, it would be very difficult to extrapolate these data on Caper without resorting to animal and clinical experiments. Moreover, the potential synergetic effect between plant bioactive molecules should be considered as well as possible drug interactions and toxicity issues.

Similarly, findings on the neuroprotective effect of Caper extracts are promising but evidenced *in vivo* studies on their use in the treatment of Alzheimer’s disease, chronic neuropathic pain, and anticholinesterases activities are lacking. Although the traditional uses of Caper include gastrointestinal and diuretic activities, no *in vitro* nor *in vivo* studies are available to date. Thus, giving scientific impetus to the traditional uses of this plant through *in vitro*, *in vivo,* and clinical studies is still needed as it has large and promising applications in disease prevention and treatment.

## Conclusions and Future Perspectives

Capers have been widely used in traditional medicine. It was reported as a good source of flavonoids, alkaloids, phenolic acids, fatty acids, and glucosinolates derivatives. Mostly, Caper is endowed with a plethora of notable biological activities mainly antibacterial, antioxidant, hepatoprotection, and anticancer. Additionally, it is an excellent candidate for the development of antidiabetic drugs. Although the large amount of literature on Caper is related to its health benefits, there are still no conclusive clinical studies regarding the association between the plant extracts/compounds and their effect on human health. Moreover, studies on the individual components of the plant are limited. Given the growing demand for natural, sustainable, and safe treatments, further studies are recommended to explore the diverse biological activities of the plant and its individual secondary metabolites both *in vitro*, *in vivo,* and through cell-based assays as well as animal studies. Noteworthily, a deep understanding of Caper chemotypes and cultivars would be essential for the selection of high-quality Caper genotypes that may be of interest for further pharmaceutical studies. Considering the phytochemical constituents and the data reported in this review, we recommend the bioprospection of Caper as a promising source of bioactive molecules to be tested in clinical experiments to evaluate their biosafety and clinical efficacy in modern pharmaceutical applications. Subsequently, Caper-based formulations should be characterized and tested with respective purposes.
