# Supplementary material for: Caper (Capparis spinosa L.): An Updated Review on Its Phytochemistry, Nutritional Value, Traditional Uses, and Therapeutic Potential
Source: Front Pharmacol. 2022 Jul 22;13:878749. doi: 10.3389/fphar.2022.878749 (PMC9353632; doi:10.3389/fphar.2022.878749)
Supplement: Supplementary file 1 [file DataSheet1.pdf]

Caper (*Capparis spinosa* L.)*:* An updated review on its phytochemistry, nutritional value, traditional uses, and therapeutic potential

Table 1S. *In vitro* antibacterial and antifungal effects of Caper extracts.

Bacteria	Plant part	Extract	Method	Key results	References
Escherichia coli	Stem	Ethanolic (80%)	Microbroth dilution	MIC=12,5 mg/mL	(1)
		Aqueous		MIC=25 mg/mL	
	Leaves	Ethanolic (80%)		MIC=6,25 mg/mL	
		Aqueous		MIC=6,25 mg/ mL	
	flowers fruits	Ethanolic (75%) (phenols)	Microbroth dilution	MIC=50 mg/mL, IZ=19 mm<br>MIC=50 mg/mL, IZ=15 mm	(2)
	Roots	Aqueous	Disc diffusion	6.50, 8.75, 10.34, 12.20, 13.42, 14.76 mm corresponding respectively to the concentrations 0.5, 1, 1.5, 2, 4, 6 mg/disc	(3)
		Methanolic (70%)		8.76, 10.45, 12.18, 14.56, 15.20, 16.56 mm corresponding respectively to the concentrations 0.5, 1, 1.5, 2, 4, 6 mg/disc	
		Ethanolic (70%)		9.20, 11.78, 13.85, 15.20, 17.36, 18.36 mm corresponding respectively to the concentrations 0.5, 1, 1.5, 2, 4, 6 mg/disc	
		Ethyl acetate (70%)		9.86, 13.30, 14.88, 17.40, 18.54, 19.72 corresponding respectively to the following crude extract 0.5, 1, 1.5, 2, 4, 6 mg/disc	
	Leaves	Petroleum ether (subsequently fractioned and gave to 2 compounds)	Broth microdilution	MIC compound 1= 100mg/mL<br>MIC compound 2= 100 mg/mL	(4)
	Flower buds	Methanolic	Disc diffusion	IZ= 21 mm	(5)
	Leaves	Ethanolic extract 80%	Agar diffusion method	NT	(6)
		Chloroform fraction		Inactive	
		Ethyl acetate fraction		IZ= 18.7 mm at 100 µl/disc<br>IZ= 16.9 mm 50 µl/disc<br>IZ= 14.9 mm at 25 µl/disc	
		N-butanol fraction		IZ= 16.3 mm at 100 µl/disc	

				IZ= 12.9 mm at 50 µl/disc<br>IZ= 9.9 mm at 25 µl/disc<br>MIC= 12.5 mg/mL	
	Flowers buds	10% decoctate	Microdilution method	MIC= 66.66 mg/mlL	(7)
				MBC= NA	
		Ethanolic		MIC= 10,26 mg/mL	
				MBC= 41,04 mg/mL	
	fruits	10% decoctate		MIC= 41.66 mg/mL	
				MBC= 66.66 mg/mL	
		Ethanolic		MIC=6.25 mg/mL	
				MBC= 8.33 mg/mL	
	fruits	Copper nanoparticles from aqueous extract	Broth microdilution	MBC=10 mg/mL	(8)
	Stems	Methanolic 80%	Disc diffusion<br><br>- Resazurin microtitre-plate assay	IZ=17.9 mm, MIC= 184.6 µg/mL	(9)
		Ethanol 80%		IZ= 13.2 mm, MIC=236.2 µg/mL	
		Acetone 80%		IZ=18.6 mm, MIC= 278.9 µg/mL	
	roots	Methanolic 80%		IZ=23.9 mm, MIC=142.6 µg/mL	
		Ethanol 80%		IZ=19.2mm, MIC=194.1 µg/mL	
		Acetone 80%		IZ=16.7mm, MIC= 229.6 µg/mL	
	fruits	Methanolic 80%		IZ=20.9mm, MIC=253.2 µg/mL	
		Ethanol 80%		IZ=15.2 mm, MIC=266.7 µg/mL	
		Acetone 80%		IZ=16.7 mm, MIC=306.9 µg/mL	
	Shoots	Methanolic (80%)		IZ= 21.9 mm, MIC= NA µg/mL	
		Ethanol (80%)		IZ= 13.7 mm MIC= NA µg/mL	
		Acetone (80%)		IZ=14.5 mm MIC= NA µg/mL	
	Flowers	Methanolic 80%		IZ=26.5 mm MIC= 181.2 µg/mL	
		Ethanol 80%		IZ=18.2 mm MIC=215.6 µg/mL	
		Acetone 80%		IZ= 21.6mm MIC=224.0 µg/mL	
	leaves	Acetone	Agar-well diffusion	IZ= 11mm	(10)
		Ethyl acetate		IZ= 13mm	
		Boiled water		IZ= 11mm	
Staphylococcus aureus	Stem	Ethanolic (80%)	Microbroth dilution	MIC=25 mg/mL	(1)
		Aqueous		MIC=25 mg/mL	
	Leaves	Ethanolic (80%)		MIC=12,5 mg/mL	
		Aqueous		MIC=12,5 mg/mL	
	flowers fruits	Ethanolic (75%) (phenols)	Microbroth dilution	MIC=50 mg/mL IZ=14 mm<br>MIC=50 mg/mL IZ=12 mm	(2)

	Twigs	Methanolic (85%)	disc diffusion	-IZ= 25.12, 21.15, 15.17, 10.23 mm corresponding respectively to the dilutions of 1 g/ml, 1, ½, ¼ ⅛	(11)
	Flower			IZ= 20.22, 15.55, 10.22, 8.75 mm corresponding respectively to the dilutions of 1 g/ml, 1, ½, ¼, ⅛	
	Fruits			IZ= 17.12, 12.50, 10.13, 7.75 mm corresponding respectively to the dilutions of 1 g/ml, 1, ½, ¼, ⅛	
	Leaves			IZ= 22.02, 15.50, 10.13, 8.78 mm corresponding respectively to the dilutions of 1 g/ml, 1, ½, ¼, ⅛	
	Roots			IZ= 11.83, 11.42, 9.83, 7.11 mm corresponding respectively to the dilutions of 1 g/ml, 1, ½, ¼, ⅛	
	Leaves	Petroleum ether (subsequently fractioned and gave to 2 compounds)	Broth microdilution	MIC compound 1= 100 mg/mL<br>MIC compound 2= 200 mg/mL	(4)
	Flower buds	Methanolic e	Disc diffusion	IZ= 22 mm	(5)
	Leaves	Ethanolic (80%)	Agar diffusion	NT	(6)
		Chloroform fraction		Inactive	
		Ethyl acetate fraction		IZ= 16.1 mm at 100 µl/disc<br>IZ= 14.6 mm 50 µl/disc<br>IZ= 14.9 mm at 25 µl/disc<br>MIC= 25mg/ml	
		N-butanol fraction		IZ= 10.1 mm at 100 µl/disc<br>IZ= 9.1 mm at 50 µl/disc<br>MIC= 50 mg/mL	
	fruits	Copper nanoparticles	Broth microdilution	MBC=5 mg/mL<br>MIC= 5 mg/mL	(8)
	Stems	Methanolic 80%	- Disc diffusion<br><br>- Resazurin microtitre-plate assay	IZ=15.7 mm MIC= 145.2 µg/mL	(9)
		Ethanol 80%		IZ= 11.1 mm MIC=189.1 µg/mL	
		Acetone 80%		IZ=13.2 mm MIC= 256.7 µg/mL	
	Roots	Methanolic 80%		IZ=21.1 mm MIC=156.7 µg/mL	
		Ethanol 80%		IZ=14.9 mm MIC=198.7 µg/mL	
		Acetone 80%		IZ=15.6 mm MIC= 277.1 µg/mL	
	fruits	Methanolic 80%		IZ=17.7 mm MIC=110.9 µg/mL	

		Ethanol 80%		IZ=11.9 mm MIC=125.6 µg/mL	
		Acetone 80%		IZ=12.6 mm MIC=272.7 µg/mL	
	Shoots	Methanolic 80%		IZ= 21.0 mm MIC= NA µg/mL	
		Ethanol 80%		IZ= 17.9 mm MIC= NA µg/mL	
		Acetone 80%		IZ=11.1 mm MIC= NA µg/mL	
	Flowers	Methanolic 80%		IZ=23.7 mm MIC= 122.1 µg/mL	
		Ethanol 80%		IZ=14.8 mm MIC=147.8 µg/mL	
		Acetone 80%		IZ= 12.1 mm MIC=266.7 µg/mL	
	leaves	Acetone	Agarwell diffusion	IZ= 19 mm	(10)
		Ethanolic		IZ= 15 mm	
		Ethyl acetate		IZ= 13 mm	
		Petroleum ether		IZ= 16 mm	
		Methanolic		IZ= 19 mm	
		Boiled water		IZ= 12 mm	
Staphylococcus epidermidis	Leaves	Petroleum ether (subsequently fractioned and gave to 2 compounds)	broth microdilution	MIC compound 1= 100mg/mL MIC compound 2= 200mg/mL	(4)
Sarm (staphylococcus aureus resistant to methicillin)	Stem	Ethanolic (80%)	microbroth dilution	MIC=6.25-12.5 mg/mL	(7)
		Aqueous		MIC=100 mg/mL	
	Leaves	Ethanolic (80%)		MIC=12,5 mg/mL	
		Aqueous		MIC=50 mg/mL	
	Flowers buds	10% decoctate	Microdilution	MIC= 66.66 mg/mL, MBC= NA	
		Ethanolic		MIC= 14.06 mg/mL, MBC= 36.04 mg/mL	
	fruits	10% decoctate		MIC= 33.33 mg/mL, MBC= NA	
		Ethanolic		MIC=8.33 mg/mL, MBC= 18.38 mg/mL	
	leaves	Acetone	Agarwell diffusion	IZ= 16 mm	(10)
		Ethanolic		IZ= 13 mm	
		Ethyl acetate		IZ= 18 mm	
		Petroleum ether		IZ= 15 mm	
		Methanolic		IZ= 13 mm	
		Boiled water		IZ= 15 mm	
Sasm (staphylococcus aureus sensitive to methicillin)	Flowers buds	10% decoctate	Microdilution	MIC= 66.66 mg/mL, MBC= NA	(7)
		Ethanolic		MIC= 12.5 mg/mL, MBC= 40 mg/mL	
	fruits	10% decoctate		MIC= 33.33 mg/mL, MBC= NA	
		Ethanolic		MIC=21.62 mg/mL, MBC= 38.29	

				mg/mL	
Pseudomonus aeruginosa	Flowers	Ethanolic (75%) (phenols)	microbroth dilution	MIC=50 mg/mL	(2)
	Fruits			MIC=50 mg/mL	
	Flower buds	Methanolic	Disc diffusion	IZ= 19 mm	(5)
	Leaves	Ethanolic 80%	Agar diffusion	NT	(6)
		Chloroform fraction		Inactive	
		Ethyl acetate		IZ= 18.1 mm at 100 µl/disc<br>IZ= 15.9 mm 50 µl/disc<br>IZ= 10.1 mm at 25 µl/disc<br>MIC= 25 mg/ml	
		N-butanol fraction		IZ= 14.9 mm at 100 µl/disc<br>IZ= 12.1 mm at 50 µl/disc<br>IZ= 10.1 mm at 25 µl/disc<br>IZ= 9.9 mm at 12.5 µl/disc<br>MIC= NA mg/ml	
	Flowers buds	Decoction	Microdilution method	MIC= NA	(7)
				MBC= NA	
		Ethanolic extract		MIC= 12.76 mg/mL	
				MBC= NA	
	fruits	Decoction		MIC= 33.33 mg/mL	
				MBC= NA	
		Ethanolic extract		MIC=NA	
				MBC= NA	
	Aerial parts	Methanolic extract	Disc diffusion	IZ= 30 mm	(12)
	leaves	Ethyl acetate	Agar-well diffusion	IZ= 14 mm	(10)
Klebsiella pneumonia	Stem	Ethanolic (80%)	microbroth dilution	MIC=50 mg/mL	(1)
		Aqueous		MIC=100 mg/mL	
	Leaves	Ethanolic (80%)		MIC=25 mg/mL	
		Aqueous		MIC=50 mg/mL	
	Leaves	Petroleum ether (subsequently fractioned and gave to 2 compounds)	broth microdilution	MIC compound 1= 50 mg/mL<br>MIC compound 2= 200mg/mL	(4)
	Flower buds	Methanolic	Disc diffusion	IZ= 16 mm	(5)
	Flowers buds	10% decoctate	Microdilution	MIC= NA	(7)
				MBC= NA	
		Ethanolic		MIC= 13.5 mg/mL	
				MBC= 50 mg/mL	

	fruits	10% decoctate		MIC= 55.55 mg/mL	
				MBC= NA	
		Ethanolic		MIC= 32.44 mg/mL	
				MBC= 86.5 mg/mL	
	fruits	Copper nanoparticules	Broth microdilution	MBC= 10 mg/mL	(8)
				MIC= 10 mg/mL	
	leaves	Acetone	Agar-well diffusion	IZ= 12 mm	(10)
		Ethanolic		IZ= 17 mm	
		Ethyl acetate		IZ= 36 mm	
		Petroleum ether		IZ= 14 mm	
		Methanolic		IZ= 15 mm	
		Boiled water		IZ= 15 mm	
Klebsiella oxytoca	Flower buds	Methanolic	Disc diffusion	IZ= 19 mm	(5)
Klebeslla sp	flowers	Ethanolic (75%) (phenols)	microbroth dilution	MIC=50 mg/mL; IZ=20 mm	(2)
	fruits			MIC=50 mg/mL; IZ=15 mm	
Bacillus subtilis	flowers	-Ethanolic (75%) (phenols)	Microbroth dilution	MIC=50 mg/m; IZ=12 mm	(2)
	fruits			MIC=50 mg/mL; IZ=13 mm	
	Leaves	-Ethanolic 80%	Agar diffusion	NT	(6)
		Chloroform		IZ= 13.3 mm at 100 µl/disc<br>IZ= 10.3 mm 50 µl/disc<br>MIC= 25 mg/ml	
		Ethyl acetate fraction		IZ= 19.8 mm at 100 µl/disc<br>IZ= 18.2 mm at 50 µl/disc<br>IZ= 15.3 mm at 25 µl/disc<br>MIC= 25 mg/mL	
		N-butanol fraction		inactive	
	Stem	Methanolic 80%	- Disc diffusion<br>- Resazurin microtitre-plate assay.	IZ=26.8 mm MIC= 123.2 µg/mL	(9)
		Ethanol 80%		IZ= 19.2 mm MIC=159.7 µg/mL	
		Acetone 80%		IZ=13.7 mm MIC= 207.8 µg/mL	
	roots	Methanolic 80%		IZ=21.7 mm MIC=157.8 µg/mL	
		Ethanol 80%		IZ=16.1 mm MIC=231.4 µg/mL	
		Acetone 80%		IZ=17.7 mm MIC= 282.6 µg/mL	
	fruits	Methanolic 80%		IZ= 23.9 mm MIC=126.7 µg/mL	
		Ethanol 80%		IZ= 16.1 mm MIC=160.1 µg/mL	
		Acetone 80%		IZ=12.7 mm MIC=253.9 µg/mL	
	Shoots	Methanolic 80%		IZ= 24.6 mm MIC= NA µg/mL	

		Ethanol 80%		IZ= 19.9 mm MIC= NA µg/mL	
		Acetone 80%		IZ= 14.9 mm MIC= NA µg/mL	
	Flowers	Methanolic 80%		IZ=17.6 mm MIC= 111.2 µg/mL	
		Ethanol 80%		IZ=19.7 mm MIC=175.6 µg/mL	
		Acetone 80%		IZ= 11.2 mm MIC=196.7 µg/mL	
Acinetobacter	Leaves	Ethanolic 95%	broth microdilution	MIC= 50ppm MBC= 25ppm	(13)
	Fruits			MIC= 25ppm MBC= 12.5ppm	
	Stems			MIC= 50ppm MBC= 50ppm	
	Leaves	Ethyl acetate		MIC 50ppm MBC: 25ppm	
	Fruits			MIC= 12.5ppm MBC= 6.25ppm	
	Stems			MIC= 50ppm MBC= 25ppm	
Acinetobacter baumannii	Flower buds	Methanolic	Disc diffusion	IZ= 21 mm	(5)
	Aerial part	Methanolic	disc diffusion	IZ= 11 mm	(12)
Salmonella Typhi	Leaves	Petroleum ether (subsequently fractioned and gave to 2 compounds)	broth microdilution	MIC compound 1= 100 mg/mL<br>MIC compound 2= 100mg/mL	(4)
Enterobacter aerogenes	Flower buds	Methanolic	Disc diffusion	IZ= 26 mm	(5)
Listeria monocytogenes	Flowers buds	10% decoctate	Microdilution	MIC= 23.6 mg/mL, MBC= NA	(7)
		Ethanolic		MIC= 13.59 mg/mL, MBC= 42.8 mg/mL	
	Fruits	10% decoctate		MIC= 38.88 mg/mL, MBC= NA	
		Ethanolic		MIC= 1.73 mg/mL, MBC= 8.33 mg/mL	
Pasteurella multocida	Stems	Methanolic 80%	- Disc diffusion<br>- Resazurin microtitre-plate assay	IZ=24,7 mm MIC= 126.7 µg/mL	(9)
		Ethanol 80%		IZ= 12.7 mm MIC=158.3 µg/mL	
		Acetone 80%		IZ=15.3 mm MIC= 246.7 µg/mL	
	Roots	Methanolic 80%		IZ=20.4 mm MIC=207.9 µg/mL	
		Ethanol 80%		IZ=13.7 mm MIC=231.4 µg/mL	
		Acetone 80%		IZ=15.1 mm MIC= 264.8 µg/mL	
	Fruits	Methanolic 80%		IZ= 24.9 mm MIC=120.1 µg/mL	
		Ethanol 80%		IZ= 17.7 mm MIC=176.7 µg/mL	
		Acetone 80%		IZ=12.1 mm MIC=234.9 µg/mL	
	Shoots	Methanolic 80%		IZ= 23.6 mm MIC= NA µg/mL	
		Ethanol 80%		IZ= 16.9 mm MIC= NA µg/mL	
		Acetone 80%		IZ= 12.5 mm MIC= NA µg/mL	
	Flowers	Methanolic 80%		IZ=20.7 mm, MIC= 146.7 µg/mL	

		Ethanol 80%		IZ=15.9 mm, MIC=158.9 µg/mL	
		Acetone 80%		IZ= 14.7 mm, MIC=206.7 µg/mL	
Enterococcus faecalis	Flower buds	Methanolic	Disc diffusion	IZ= 22 mm	(5)
Citrobacter freundii				IZ= 17 mm	
Proteus mirabilis				IZ= 27 mm	
Proteus vulgaris				IZ= 17 mm	
Bacillus cereus	Fruits	Aqueous (Copper nanoparticles)	Broth microdilution	MBC= 5 mg/mL, MIC= 5 mg/mL	(8)
Enterococcus faecalis	Leaves	Acetone	Agarwell diffusion	IZ= 14 mm	(10)
		Ethanolic		IZ= 17 mm	
		Ethyl acetate		IZ= 29 mm	
		Petroleum ether		IZ= 11 mm	
		Methanolic		IZ= 18 mm	
		Boiled water		IZ= 12 mm	
Alcaligenes faecalis		Acetone		IZ= 20 mm	
		Ethanolic		IZ= 21 mm	
		Ethyl acetate		IZ= 17 mm	
		Petroleum ether		IZ= 14 mm	
		Methanolic		IZ= 16 mm	
		Boiled water		IZ= 12 mm	
Enterobacter cloacae		Acetone		IZ= 17 mm	
		Ethanolic		IZ= 19 mm	
		Ethyl acetate		IZ= 19 mm	
		Petroleum ether		IZ= 13 mm	
		Methanolic		IZ= 14 mm	
		Boiled water		IZ= 16 mm	

In vitro antifungal effects of Caper extracts.

Fungi	Plant part	Extract	Method	Key results	References
Candida albicans	Leaves	Ethyl acetate	Agar diffusion and microdilution	IZ= 10.2 – 16.8 mm<br>MIC= 25 mg/ml	(6)
		N- Butanol		IZ= 9.9-10 mm<br>MIC= 50 mg/mL	
		Ethyl acetate	Microdilution	MIC ≤ 12.5 µg/mL	(10)
			Agar diffusion	IZ= 17 mm	
		Ethanolic	Microdilution	MIC ≤ 12.5 µg/mL	
			Agar diffusion	IZ=19 mm	
		Methanolic	Microdilution	MIC ≤ 12.5 µg/mL	

			Agar diffusion	IZ= 14 mm	
	Fruits	Aqueous (Silver nanoparticles)	broth microdilution	MIC=2500µg/mL	(14)
Aspergillus flavus.	Twings	Methanolic (85%)	Agar diffusion	IZ= 8.43. 7.12. and 7.73 mm correspond respectively to the following concentrations 1g/mL; 500 mg/ mL; 250 mg/mL	(11)
Candida glabrata	Fruits	Aqueous (Silver nanoparticles)	broth microdilution	MIC= 5000µg/mL	(14)
				MBC= 10000µg/mL	
Kluyveromyces marxianus				MIC= 625µg/mL	
				MBC= 156.25µg/mL	
Fusarium moniliforme	Leaves	Ethyl acetate	Agar well diffusion	IZ= 21 mm	(10)
Trichoderma longibrachiatum		Ethyl acetate		IZ=16 mm	
		Boiled water		IZ= 17 mm	
Trichoderma viride		Ethyl acetate		IZ= 19 mm	
Penicillium aurantiacum		Ethyl acetate		IZ=18 mm	
Aspergillus niger		Methanolic		IZ=16 mm	
Rhizopus stolonifer		Ethyl acetate		IZ=25 mm	
Curvulariacl avata		Acetone		IZ=12 mm	
		Ethanolic		IZ=17 mm	
		Ethyl acetate		IZ=25 mm	
		Pet. Ether		IZ=17 mm	
		Methanolic		IZ=15 mm	
		Boiled water		IZ=13 mm	

IZ: Inhibition zone. MIC: Minimum inhibitory concentration. MBC: Minimum bactericidal concentration

Table 2S. *In vitro* antioxidants effects of Caper extracts.

Plant part	Collection zone	Extraction method	Extract	Method	Key results	Reference
Leaves	Southern part of Iran (Ewaz of Fars province)	Percolation	Hydro-ethanolic	DPPH	IC_{50}=1.41 mg/ml	(15)
Fruits					IC_{50}= 1.56 mg/ml	
Buds					IC_{50}= 2.49 mg/ml	
Leaves	Jahrom city, Fars province, Iran	Maceration	Hydro-ethanolic 70%	DPPH	IC_{50}= = 3624 ppm	(16)
Fruits					IC_{50}= 21591 ppm	
Aerial part	Cholistan desert	Maceration	Methanolic	DPPH	30.48± 0.37 mg TE/g extract	(17)
				ABTS	40.43 ± 3.33 mg TE/g extract	
				FRAP	47.13 mg TE/g extract	
				CUPRAC	86.64 mg TE/g extract	
				Phosphomolybdenum assay	6.73 mg TE/g extract	
				Metal chelating power	1.19 mg EDTA/g	
			Dichloromethane	DPPH	6.24 TE/g extract	
				ABTS	23.64 mg TE/g extract g	
				FRAP	50.37 mg TE/g extract	
				CUPRAC	118.45 mg TE/g extract	
				Phosphomolybdenum assay	75.79 mg TE/g extract	
				Metal chelating power	2.51 mg EDTA/	
Roots			Methanolic	DPPH	28.45 mg TE/g extract	
				ABTS	40.55mg TE/g extract	
				FRAP	38.49 mg TE/g extract	
				CUPRAC	58.77 mg TE/g extract	
				Phosphomolybdenum assay	NA mg TE/g extract	
				Metal chelating power	0.31 mg EDTA/g	
			Dichloromethane	DPPH	16.06 mg TE/g extract	
				ABTS	33.68 mg TE/g extract	
				FRAP	42.82 mg TE/g extract	
				CUPRAC	96.89 mg TE/g extract	
				Phosphomolybdenum assay	13.56 mg TE/g extract	

				Metal chelating power	1.41 mg EDTA/g	
Flower buds (small, medium and big), sprout and fruits	Mersin, Turkey	Not specified	Methanolic	DPPH	Antioxidant activity (%):<br>Bud (small)= 74.62 %<br>Bud (medium)= 76.65 %<br>Bud (big)= 72.21 %<br>Sprout=59.01 %<br>Fruit=50.51 %	(18)
Leaves	different regions in southern Tunisia	Maceration	Aqueous	DPPH	IC_{50}= 41.57 mg/mL	(19)
				ABTS	206.07 mg EAA/g DW	
				FRAP	EC_{50}= 120.22 mg/mL	
		Reflux		DPPH	IC_{50}= 36.66 mg/mL	
				ABTS	179.68 mg EAA /g DW	
				FRAP	EC_{50}= 89.91 mg/mL	
		Ultrasonic assisted extraction.		DPPH	IC_{50}= 40.20 mg/mL	
				ABTS	258.77 mg EAA /g DW	
				FRAP	EC_{50}= 62.07 mg/mL	
Aerial parts	Botraba, Libya	Cold maceration	Methanolic (70%)	DPPH	% inhibition = 0.07 %	(20)
Fruit	Khuzestan Province, Iran	Electric mixer	Ethanolic (70%)	DPPH	EC_{50}=1.48 mg/mL	(21)
Roots	Anbarabad, Iran	Ultrasound	Ethanolic (70%)	DPPH	IC_{50}=79.28 µg/mg	(22)
			Aqueous		IC_{50}= 90.09 µg/mg	
Flower buds	Alicante, Almería and Murcia, Spain	Sonication	Methanolic (80%)	DPPH	1542.5 ± 180.4 mg Trolox/100 g FW	(23)
				FRAP	160.2 ± 17.0 mg Trolox/100 g FW	
				ABTS	192.7 ± 0.8 mg Trolox/100 g FW	
Leaves	Albaha, Saudi Arabia	Infusion	Ethanolic (80 %)	DPPH	IC_{50}= 0.0707 mg/mL, Activity % = 61.00 %	(24)
			n-Butanol		IC_{50}= 0.043 mg/mL, Activity % = 68.55 %	
Fresh flower buds	Gaziantep province, Turkey	Magnetic stir	Methanolic (75%)	DPPH	51.25 µM Trolox/kg	(25)
				ABTS	62.19 µM Trolox/kg	
Fermented flower buds				DPPH	21.41 µM Trolox/kg	
				ABTS	32.98 µM Trolox/kg	
Processed and fresh fruit	Fez, Morocco	Not mentioned	Acetone (80%)	DPPH	Processed buds IC_{50}=8.18 µg/ml<br>Small fresh buds IC_{50}=5.90 µg/ml	(26)
Leaves	Archipelago, Italy	Decoction for leaves	Methanolic 60%	DPPH	38.82 mg TE/g extract	(27)
				ABTS	96.25 mg TE/g extract	
				CUPRAC	80.77 mg TE/g extract	
				FRAP	56.33 mg TE/g extract	
				Phosphomolybdenum assay	0.37 mmol TE/g extract	

				Metal chelating power	4.36 mg EDTAE/g extract	
Aerial parts		Microwave extraction		DPPH	29.33 mg TE/g extract	
				ABTS	86.78 mg TE/g extract	
				CUPRAC	73.47 mg TE/g extract	
				FRAP	52.67 mg TE/g extract	
				Phosphomolybdenum assay	0.50 mmol TE/g extract	
				Metal chelating power	3.01 mg EDTAE/g extract	
		Soxhlet extraction		DPPH	60.54 mg TE/g extract	
				ABTS	124.15 mg TE/g extract	
				CUPRAC	98.17 mg TE/g extract	
				FRAP	66.94 mg TE/g extract	
				Phosphomolybdenum assay	0.56 mmol TE/g extract	
				Metal chelating power	2.34 mg EDTAE/g extract	
Aerial parts	South Sinai region, Egypt	Soxhlet	Petroleum ether	DPPH	-DPPH activity: EC_{50}= 17.66 mg extract/ mg<br>Antioxidant efficiency AE= 0.056	(28)
				ABTS	Inhibition = 27.54%	
				FIC	IC_{50}= 0.662 mg extract/ mL	
				FRAP	225.0 mmol Fe(II)/ mg extract	
Leaves, flowers, fruits, roots	Siliana, Tunisia	Maceration	Methanolic	TAC	Leaves 112.4 mg EAA / g of dry extract<br>Flowers 67.3 mg EAA / g of dry extract<br>Fruits 97 mg EAA / g of dry extract<br>Roots 175.7 mg EAA / g of dry extract.	(29)
				DPPH	Leaves: IC_{50} = 70.1 mg/L<br>Flowers: IC_{50} = 137.1 mg/L<br>Fruits: IC_{50} = 172.5 mg/L<br>Roots: IC_{50} = 329 mg/L	
Leaves	Khuzestan, Iran	Maceration	-Ethanol 80%<br>-ethyl acetate<br>-chloroform	DPPH	Hydro-alcoholic: IC_{50}= 0.034891 mg/ml<br>Ethyl acetate: IC_{50}= 0.277039 mg/ml<br>Chloroform: IC_{50}= 0.0392 mg/ml	(30)
				FRAP	Hydro-alcoholic 1.655187 mg/ml<br>Ethyl acetate 15.90301 mg/ml<br>Chloroform 3.908373 mg/ml	

Flower buds and fruits	Sidi Kacem, Morocco	Maceration	Ethanolic	DPPH	IC_{50}= 0.52 mg/ml for flower buds and 0.61 mg/ml for fruits.	(31)
		Decoction	Aqueous		IC50= 0.25 mg/ml for flower buds and 0.4 mg/ml for fruits.	
Fruit	Jaén, Spain	ultrasound-assisted solid-liquid extraction	Methanolic	DPPH	Fruits: 0.98 g TE/100 g extract<br>Laboratory fermented fruits: 1.08 g TE/100 g extract<br>Commercially fermented fruits: 1.48 g TE/100 g extract	(32)
				ABTS	Fruits: 1.72 g TE/100 g extract<br>Laboratory fermented fruits: 1.92 g TE/100 g extract<br>Commercially fermented fruits: 2.21 g TE/100 g extract	
Leaves	Al Bayda city, Libya	Soxhlet	Methanolic	DPPH	IC_{50} = 0.205 mg/ml	(33)
Seeds	South Sinai Governorate, Egypt	Soxhlet	Petroleum ether	DPPH	%= 152.40 %	(34)
Leaves	Gafsa, Tunisia	Not specified	Methanolic	DPPH	EC_{50} = 43.031 ± 1.24 µg/ml	(35)
				H_2O_2	EC_{50} = 81.21 ± 1.28 µg/ml	
Fruits	Not mentioned	Not specified	Methanol, ethanol, aqueous	DPPH	Ethanol: 38,8% as the highest activity.	(36)
Leaves	Kazeroon city, Iran	Reflux with deionized water	Polysaccharides	DPPH	DPPH activity % = 55% at 300 µg/mL	(37)
				Hydroxyl (OH) scavenging activity	OH activity % = 64% at 300 µg/mL	
Aerial parts	Amman, Jordan	Infusion	Ethanolic (70%)	DPPH	IC_{50}= 76 µg/ml	(38)
				FRAP	0.5 absorbance is achieved at 5.59 mg/mL	
			Aqueous	DPPH	IC_{50}= 209 µg/ml	
				FRAP	0.5 absorbance is achieved at 13.0 mg/mL	
Leaves	Tunisia	Soxhlet	Ethanolic	DPPH	IC_{50}= 31.73 µg/ml	(39)
				ABTS	C_{50}=34.02 µg/ml	
			Aqueous	DPPH	IC_{50}= 45.62 µg/ml	
				ABTS	IC_{50}=38.32 µg/mL	
Not specified	Not indicated	Maceration	Ethanolic (80%)	DPPH	IC_{50} = 7.41 µg/mL	(40)
				ABTS	3.15 TEAC	
Roots	Abu-graib, Iraq	reflux for aqueous extract	Aqueous	FRAP	74.29% at 10 mg/mL	(41)
				Chelating ability	11.35% and 51.50% at 2 mg/mL and 10 mg/mL	

		Maceration for ethanolic extract	Ethanolic (80%)	FRAP	Frap activity: 67.33% at 10 mg/mL	
				Chelating ability	9.02% and 35.21% 2 mg/mL and 10 mg/mL	
Fruits	Tunisia	Not specified	Methanolic	TAC	The highest activity is: 78.63 mg GAE/g dried residue	(42)
				DPPH	The highest IC_{50} is 126.5 µg/mL	
				ABTS	ABTS activity: the highest ic50 is 74.82 µg/mL	
Not indicated	Gaziantep, Turkey	Sonication	Methanolic	FRAP	145.07 µmol Trolox.100 g-1 dw	(43)
				DPPH	SC_{50}= 0.32 mg. ml-1	

Trolox equivalent; EDTAE: EDTA equivalent; NA: not active, EAA: equivalents ascorbic acid, FRAP assay: Equivalent concentration 1 EC1) mmol/L FeSO4

Table 3S. Physicochemical parameters of caper phytoconstituents.

Compound	MW	Hydrogen bond acceptors	Hydrogen bond donors	Consensus Log P	Lipinski	Ghose	Veber	Egan	Muegge
					Violations				
**Alkaloids**									
6	143.18	2	0	-1.59	0	2	0	0	1
7	161.16	2	2	1.24	0	1	0	0	1
8	156.18	1	1	1.83	0	1	0	0	1
9	496.46	12	8	-2.46	2	2	1	1	4
10	205.21	3	2	1.78	0	0	0	0	0
11	207.18	4	3	0.34	0	0	0	0	0
12	145.16	1	1	1.72	0	2	0	0	1
13	161.16	2	2	1.56	0	1	0	0	1
14	278.26	4	2	2.4	0	0	0	0	0
15	214.22	6	4	-1.18	0	1	0	0	1
16	435.52	5	4	2.33	0	1	0	0	0
17	465.54	6	4	2.38	0	1	0	0	0
18	465.54	6	4	2.35	0	1	0	0	0
19	465.54	6	4	2.33	0	1	0	0	0
20	435.52	5	4	2.34	0	1	0	0	0
21	597.66	10	7	0.47	3	4	1	1	2
**Glucosinolates**									
22	597.66	10	7	0.47	3	4	1	1	2

23	448.47	10	6	-0.3	2	0	1	1	2
24	478.49	11	5	-0.08	1	0	1	1	2
25	478.49	11	6	-0.29	2	0	1	1	3
26	464.47	11	7	-0.89	2	0	1	1	3
27	221.28	2	1	2.23	0	0	0	0	0
28	280.37	3	1	1.83	0	0	0	0	0

29	333.34	10	5	-1.61	0	1	1	1	1
**Furans and pyrroles**									
30	234.2	5	0	1.23	0	0	0	0	0
31	126.11	3	1	0.19	0	3	0	0	1
32	142.11	4	2	0.65	0	3	0	0	1
33	139.15	2	1	0.71	0	3	0	0	1
34	132.11	4	2	-0.61	0	4	0	0	1
35	253.25	5	2	-0.33	0	1	0	0	0
36	179.17	3	0	0.7	0	0	0	0	1
37	253.25	5	2	-0.14	0	1	0	0	0
38	226.23	5	2	-0.93	0	1	0	0	0
39	237.21	5	3	-0.98	0	1	0	0	0
**Flavonoids**									
40	286.24	6	4	1.58	0	0	0	0	0
41	270.24	5	3	2.11	0	0	0	0	0
42	610.52	16	10	-1.29	3	4	1	1	4
43	1205.04	31	19	-2.63	3	4	2	1	6
44	600.53	12	6	3.5	3	2	1	1	5
45	300.26	6	3	2.11	0	0	0	0	0
46	286.28	5	2	2.25	0	0	0	0	0
47	448.38	11	7	-0.25	2	0	1	1	3
48	316.26	7	4	1.65	0	0	0	0	0
49	772.66	21	13	-3.18	3	4	1	1	5
50	610.52	16	10	-1.27	3	4	1	1	4
51	566.51	10	4	4.34	1	3	1	1	2
52	566.51	10	4	4.39	1	3	1	1	2
**Terpenoids**									
53	224.3	3	2	1.46	0	0	0	0	0
54	386.44	8	5	-0.02	0	1	0	1	0
55	402.44	9	6	-1.06	1	1	1	1	2

56	386.44	8	5	0.01	0	1	0	1	0
57	416.42	10	6	-0.88	1	1	1	1	2
58	402.44	9	5	-0.56	0	1	1	1	1
59	240.3	4	2	0.94	0	0	0	0	0
60	280.32	5	2	0.94	0	0	0	0	0
61	248.27	6	4	-0.53	0	1	0	0	0
62	194.18	6	4	-1.39	0	1	0	0	1
63	414.71	1	1	7.26	1	3	0	1	2
64	576.85	6	4	5.49	1	4	0	0	1
65	843.31	7	3	11.39	2	4	1	1	3
66	430.71	2	1	8.29	1	3	1	1	1
67	462.7	4	1	6.52	0	3	1	1	1
68	180.16	6	6	-2.81	1	2	0	0	3
69	172.18	4	0	0.62	0	0	0	0	1
70	186.16	3	0	2.21	0	0	0	0	1
71	186.16	3	0	2.12	0	0	0	0	1

Table 4S. Bioavailability Score, Fraction Csp3, topological polar surface area (TPSA), number of rotatable bonds (RB) and solubility (ESOL Log S) of caper phytoconstituents.

Compounds	Bioavailability Score	Fraction Csp3	RB	TPSA	ESOL Log S
**Alkaloids**					
6	0.55	0.86	1	40.13	-0.91
7	0.55	0	1	53.09	-2.06
8	0.55	0.1	1	39.58	-2.31
9	0.17	0.59	7	218.11	-1.01
10	0.55	0.18	2	62.32	-2.83
11	0.56	0.2	2	86.63	-1.26
12	0.55	0	1	32.86	-2.5
13	0.85	0	1	53.09	-2.58
14	0.56	0	2	79.12	-3.92
15	0.55	0.56	4	106.7	0.41
16	0.55	0.28	0	99.69	-4.75
17	0.55	0.31	1	108.92	-4.83
18	0.55	0.31	1	108.92	-4.83
19	0.55	0.31	1	108.92	-4.83
20	0.55	0.28	0	99.69	-4.75
21	0.17	0.42	3	178.84	-4.35
**Glucosinolates**					
22	0.17	0.42	3	178.84	-4.35
23	0.11	0.44	7	215.58	-2.33
24	0.11	0.47	8	213.95	-2.66
25	0.11	0.47	8	224.81	-2.42
26	0.11	0.44	7	235.81	-2.2
27	0.55	0.18	3	67.39	-2.93
28	0.55	0.33	2	101.29	-2.8
29	0.11	0.88	5	199.79	-0.44
**Furans and pyrroles**					

30	0.55	0.17	6	69.65	-1.87
31	0.55	0.17	2	50.44	-0.54
32	0.85	0.17	1	70.67	-1.72
33	0.55	0.29	3	42.09	-0.99
34	0.55	0.8	1	66.76	-0.05
35	0.55	0.58	1	80.92	-0.66
36	0.55	0.33	1	48.3	-1.48
37	0.55	0.58	2	80.92	-0.94
38	0.55	0.7	3	86.71	-0.14
39	0.55	0.3	3	114.43	-0.54
**Flavonoids**					
40	0.55	0	1	111.13	-3.31
41	0.55	0	1	90.9	-3.94
42	0.17	0.44	6	269.43	-3.3
43	0.17	0.44	12	518.63	-5.4
44	0.17	0.06	4	200.26	-7.42
45	0.55	0.06	2	100.13	-3.9
46	0.55	0.19	2	75.99	-3.7
47	0.17	0.29	4	190.28	-3.18
48	0.55	0.06	2	120.36	-3.36
49	0.17	0.55	9	348.58	-2.69
50	0.17	0.44	6	269.43	-3.3
51	0.55	0.06	5	159.8	-7.17
52	0.55	0.06	5	159.8	-7.17
**Terpenoids and miscellaneous**					
53	0.55	0.62	2	57.53	-1.45
54	0.55	0.74	5	136.68	-1.24
55	0.55	0.74	6	156.91	-0.84
56	0.55	0.74	5	136.68	-1.24
57	0.11	0.68	6	173.98	-0.9

58	0.55	0.84	5	145.91	-0.63
59	0.55	0.77	2	66.76	-0.83
60	0.55	0.6	3	83.83	-0.83
61	0.55	0.82	4	99.38	-0.68
62	0.55	1	3	99.38	0.14
63	0.55	0.93	6	20.23	-7.9
64	0.55	0.94	9	99.38	-7.7
65	0.17	0.94	27	105.45	-13.48
66	0.55	0.79	12	29.46	-8.6
67	0.55	0.86	12	63.6	-6.99
68	0.55	1	0	121.38	1.38
69	0.55	0.62	2	44.76	-1.01
70	0.55	0	0	43.35	-2.99
71	0.55	0	0	43.35	-2.73

Table 5S. Pharmacokinetics behavior of caper phytoconstituents. Compounds number according to Figures 3, 4, 5 and 6 at the main text.

Compounds	GI absorption	BBB permeant	Pgp substrate	CYP1A2	CYP2C19	CYP2C9 inhibitor	CYP2D6	CYP3A4	log Kp (cm/s)
**Alkaloids**									
6	Low	No	No	No	No	No	No	No	-6.89
7	High	Yes	No	Yes	No	No	No	No	-6.46
8	High	Yes	No	Yes	No	No	No	No	-6.12
9	Low	No	No	No	No	No	No	No	-11.17
10	High	Yes	No	No	No	No	No	No	-5.97
11	High	No	No	No	No	No	No	No	-7.6
12	High	Yes	No	Yes	No	No	No	No	-5.82
13	High	Yes	No	Yes	No	No	No	No	-5.87
14	High	No	No	Yes	No	No	Yes	No	-5.91
15	High	No	No	Yes	No	No	No	No	-9.42
16	High	No	Yes	No	No	No	No	Yes	-6.78
17	High	No	Yes	No	No	Yes	No	No	-6.99
18	High	No	Yes	No	No	No	No	No	-6.99
19	High	No	Yes	No	No	No	No	No	-6.99
20	High	No	Yes	No	No	No	No	Yes	-6.78
21	Low	No	Yes	No	No	No	No	No	-9.05
**Glucosinolates**									
22	Low	No	Yes	No	No	No	No	No	-9.05
23	Low	No	No	No	No	No	No	No	-9.1
24	Low	No	Yes	No	No	No	No	No	-9.03
25	Low	No	Yes	No	No	No	No	No	-9.3
26	Low	No	No	No	No	No	No	No	-9.45
27	High	Yes	No	Yes	Yes	No	No	No	-6
28	High	No	No	Yes	Yes	Yes	No	No	-6.77
29	Low	No	Yes	No	No	No	No	No	-9.62
**Furans and pyrroles**									

30	High	Yes	No	Yes	No	No	No	No	-7.12
31	High	No	No	No	No	No	No	No	-7.48
32	High	No	No	No	No	No	No	No	-6.38
33	High	Yes	No	No	No	No	No	No	-7.01
34	High	No	No	No	No	No	No	No	-7.72
35	High	No	No	No	No	No	No	No	-8.85
36	High	Yes	No	No	No	No	No	No	-7.05
37	High	No	No	No	No	No	No	No	-8.46
38	Low	No	No	No	No	No	No	No	-8.7
39	High	No	No	No	No	No	No	No	-8.64
**Flavonoids**									
40	High	No	No	Yes	No	No	Yes	Yes	-6.7
41	High	No	No	Yes	No	No	Yes	Yes	-5.8
42	Low	No	Yes	No	No	No	No	No	-10.26
43	Low	No	Yes	No	No	No	No	No	-15.23
44	Low	No	No	No	No	Yes	No	No	-5.92
45	High	No	No	Yes	No	No	Yes	Yes	-6.11
46	High	Yes	No	Yes	Yes	No	No	Yes	-6.02
47	Low	No	No	No	No	No	No	No	-8.52
48	High	No	No	Yes	No	No	Yes	Yes	-6.9
49	Low	No	Yes	No	No	No	No	No	-12.77
50	Low	No	Yes	No	No	No	No	No	-10.26
51	Low	No	No	No	No	Yes	No	No	-5.72
52	Low	No	No	No	No	Yes	No	No	-5.72
**Terpenoids and miscellaneous**									
53	High	Yes	No	No	No	No	No	No	-7.28
54	Low	No	Yes	No	No	No	No	No	-9.4
55	Low	No	No	No	No	No	No	No	-10
56	Low	No	Yes	No	No	No	No	No	-9.4
57	Low	No	No	No	No	No	No	No	-10.11

58	Low	No	Yes	No	No	No	No	No	-10.3
59	High	No	No	No	No	No	No	No	-8.18
60	High	No	No	No	No	No	No	No	-8.18
61	High	No	Yes	No	No	No	No	No	-8.3
62	Low	No	No	No	No	No	No	No	-8.6
63	Low	No	No	No	No	No	No	No	-2.2
64	Low	No	No	No	No	No	No	No	-4.32
65	Low	No	Yes	No	No	No	No	Yes	0.04
66	Low	No	Yes	No	No	No	No	No	-1.33
67	Low	No	Yes	No	No	No	No	No	-3.41
68	Low	No	Yes	No	No	No	No	No	-10.03
69	High	Yes	No	No	No	No	No	No	-7.08
70	High	Yes	No	Yes	No	No	No	No	-5.96
71	High	Yes	No	Yes	No	No	No	No	-6.25

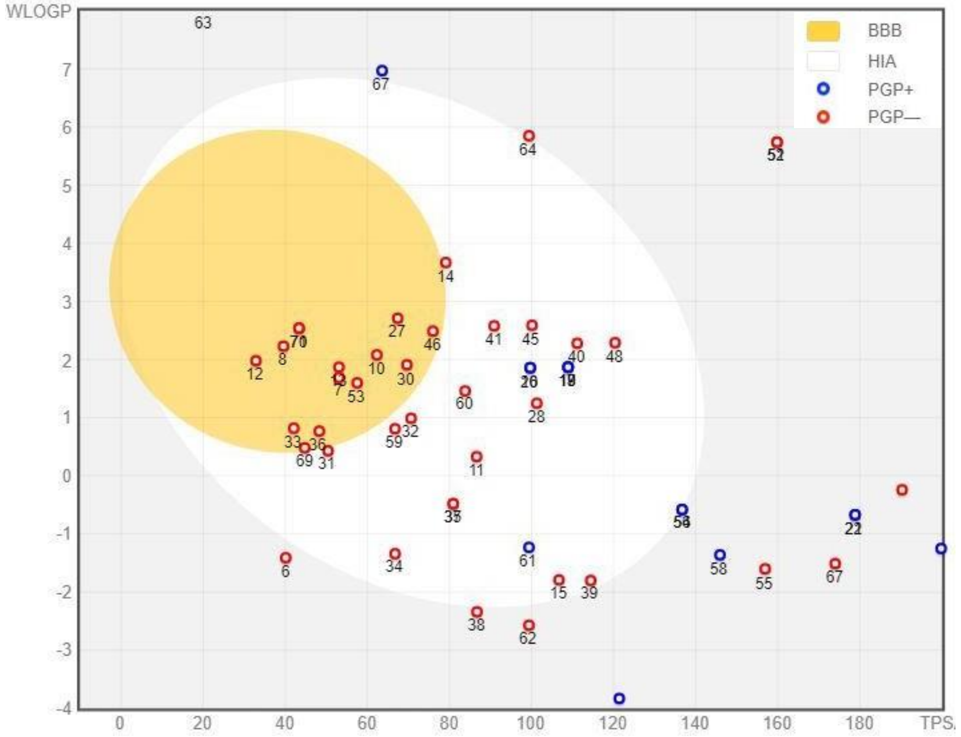

Figure 1S. Boiled-Egg of caper phytoconstituents highlighting that 14 compounds are able to cross the blood membrane barrier (BBB), 36 compounds passively absorbed by the gastrointestinal tract (HIA) and 45 compounds are not effluated from the central nervous system by the P-glycoprotein (PGP-), 12 compounds effluated from the central nervous system by the P-glycoprotein (PGP+), and 13 compounds are out of range. Compound numbers are from Table 1S.

References

1. Adwan GM, Omar GI. Evaluation of Antimicrobial activity and Genotoxic Potential of Capparis spinosa (L .) Plant Extracts. Microbiology Research Journal International. 2021;31(1):48- 57.

2. Hameed AT, Zaidan DH, Dawd SM. The Phytochemical Constituent Of Capparis Spinosa L . And Phenolic Activity On Pathogenic Bacteria And Blood Parameters. Systematic Reviews in Pharmacy. 2021;12(1):1193- 8.

3. Kumari M. In vitro evaluation of antimicrobial activity of roots extracts of capparis spinosa l. and important medicinal plant. Indian Journal of Scientific Research. 2020;(2010).

4. Gaber A, Al-saudi THA, Muraih JK. Extracted chemical compounds from Capparis spinosa leaves and their antibacterial activity on pathogenic bacteria . Journal of Pharmaceutical Sciences and Research. 2019;11(2):603- 8.

5. Mickymaray S, Saleh M, Aboody A. medicina In Vitro Antioxidant and Bactericidal E ffi cacy of 15 Common Spices : Novel Therapeutics for Urinary Tract Infections ? 2019;

6. Fadlelmula AA, Alomari AALI. Phytochemical Study and Antimicrobial Activity of Two Medicinal plants from Al-Baha region of Saudi Arabia. Oriental journal of chemistry. 2019;35:1782- 8.

7. Ennacerie Fatima-Zahra RFF, Moukrad N, Ed-Dra A. Antibacterial synergistic effect of extracts of the organs of capparis spinosa and in combination with antibiotics. International journal of advanced research. 2017;5(9):1238- 47.

8. Ebrahimi K, Shiravand S, Mahmoudvand H. Biosynthesis of copper nanoparticles using aqueous extract of Capparis spinosa fruit and investigation of its antibacterial activity. Marmara Pharmaceutical Journal. 2017;21(4):866- 71.

9. Gull T, Sultana B, Bhatti IA, Jamil A. Antibacterial Potential of Capparis spinosa and Capparis decidua Extracts. International Jo urnal of agriculture & Biology. 2015;727- 33.

10. Sherif MM, El-Shiekh HH, Elaasser MM, Elbadry M. In vitro evaluation of antimicrobial and cytotoxic effects of Caper (Capparis spinosa). Journal of Applied Sciences Research. 2013;(January).

11. Benzidane N, Aichour R, Guettaf S, Laadel N, Khennouf S. Chemical investigation, the antibacterial and antifungal activity of different parts of Capparis spinosa extracts. Journal of Drug Delivery and Therapeutics. 2020;10(5):118- 25.

12. Abou-Zeid HM, Bidak LM, Goha YM. Phytochemical screening and antimicrobial activities of some wild medicinal plants of the western mediterranean coastal region, egypt. International journal of pharmaceutical sciences and research. 2014;5(7):3072- 80.

13. Saeidi S, Javadian E, Javadian F. Anti-microbial and Anti-biofilm activity of leaf fruit and stem Capparis spinose extract against Acinetobacter baumannii . 2019;30(3):501- 6.

14. Ebrahimi K, Madani M, Ashrafi B, Shiravand S, Sepahvand A. Antifungal Properties of Silver Nanoparticles Synthe- sized From. Research In Molecular Medicine. 2019;98(916):43- 50.

15. Assadi S, Shafiee SM, Erfani M, Akmali M. Antioxidative and antidiabetic effects of Capparis spinosa fruit extract on high-fat diet and low-dose streptozotocin-induced type 2 diabetic rats. Biomedicine and Pharmacotherapy. 2021;138(March):111391.

16. Khojasteh M, Ghani A, Ghani E. In vitro effects of Capparis spinosa L . extract on human sperm function , DNA fragmentation , and oxidative stress. Journal of Ethnopharmacology. 2021;269(March 2020):113702.

17. Saleem H, Khurshid U, Sarfraz M, Ahmad I, Alamri A, Anwar S, et al. Investigation into the biological properties, secondary metabolites composition, and toxicity of aerial and root parts of Capparis spinosa L.: An important medicinal food plant. Food and Chemical Toxicology. 1 sept 2021;155:112404.

18. Ghafoor K, Juhaimi F Al, Özcan MM, Uslu N, Babiker EE, Ahmed IAM. Bioactive properties and phenolic compounds in bud, sprout, and fruit of Capparis spp. plants. Journal of Food Processing and Preservation. 1 mars 2020;44(3):e14357.

19. Yahia Y, Benabderrahim MA, Tlili N, Hannachi H, Ayadi L, Elfalleh W. Comparison of Three Extraction Protocols for the Characterization of Caper (Capparis spinosa L .) Leaf Extracts : Evaluation of Phenolic Acids and Flavonoids by Liquid Chromatography – Electrospray Ionization – Tandem Mass Spectrometry (LC – ESI – MS) . Analytical Letters. 2020;0(0):1- 12.

20. Elshibani F, Alamami A, Alshalmani S, Naili EA El. alpha-amylase inhibitory activities of Capparis spinosa L Estimation of phenolic content , flavonoid content , antioxidant properties and alpha-amylase inhibitory activities of Capparis spinosa L . Journal of pharmacognosy and phytochemistry. 2020;(January).

21. Hematian A, Nouri M, Dolatabad SS. Kashk with caper (Capparis spinosa L .) extract : quality during storage. Foods and Raw Materials. 2020;8(2):402- 10.

22. Safarzaei A, Sarhadi H, Haddad Khodaparast MH, Shahdadi F, Dashipour AR. Optimization of Aqueous and Alcoholic Extraction of Phenolic and Antioxidant Compounds From Caper (Capparis Spinosa L.) Roots Assisted by UltrasoundWaves. Zahedan Journal of Research in Medical Sciences. 22:2383- 894.

23. Grimalt M, Legua P, Hernández F, Amorós A, Almansa MS. Antioxidant Activity and Bioactive Compounds Contents in Different Stages of Flower Bud Development from Three Spanish Caper (Capparis spinosa) Cultivars. The Horticulture Journal. 2019;88(3):410- 9.

24. Fadlelmula AA, Alomari AA. Antioxidant Activity and Total Phenolic Contents of Dodonaea viscose and Capparis spinosa from Jebal Shuda in Albaha Area of Saudi Arabia. Asian Journal of Chemistry. 2019;31(January):445- 8.

25. Sonmezdag AS. Characterization of Aroma-Active Compounds , Phenolics , and Antioxidant Properties in Fresh and Fermented Capers (Capparis spinosa) by GC-MS-Olfactometry and LC-DAD-ESI-MS / MS. Journal of Food Science. 2019;84:2449- 57.

26. El amri N, Errachidi F, Bour A, Chabir R. Characterization of Moroccan raw and processed caper berries. Materials Today: Proceedings. 1 janv 2019;13:841- 9.

27. Mollica A, Stefanucci A, Macedonio G, Locatelli M, Luisi G, Novellino E, et al. Chemical composition and biological activity of Capparis spinosa L . from Lipari Island. South African Journal of Botany. 2018;

28. El-shahaby OA, El-zayat MM, El-fattah GA, El-hefny MM. Evaluation of the biological activity of Capparis spinosa var . aegyptiaca essential oils and fatty constituents as Anticipated Antioxidant and Antimicrobial Agents. Progress in Chemical and Biochemical Research. 2019;2(4):211-21.

29. Rajhi I, Dhia MT Ben, Abderrabba M, Ayadi S. Phytochemical screening , in vitro antioxidant and antibacterial activities of methanolic extracts of Capparis Spionsa L . different parts from Tunisia. Journal of Materials and Environmental Sciences. 2019;2508(3):234-43.

30. Kalantari H, Foruozandeh H, Khodayar MJ, Siahpoosh A, Saki N, Kheradmand P. Antioxidant and hepatoprotective effects of Capparis spinosa L. fractions and Quercetin on tert-butyl hydroperoxide- induced acute liver damage in mice. Journal of Traditional and Complementary Medicine. 2018;8(1):120-7.

31. Ennacerie F z, Filali FR, Moukrad N, Bouidra M, Bentayeb A. Evaluation of the Antioxidant Activity and the Cytotoxicity of Extracts of Capparis spinosa. International Journal of Pharmaceutical Sciences and Drug Research. 2018;10(2):57-64.

32. Jiménez-López J, Ruiz-Medina A, Ortega-Barrales P, Llorent-Martínez EJ. Phytochemical profile and antioxidant activity of caper berries (Capparis spinosa L.): Evaluation of the influence of the fermentation process. Food Chemistry. 1 juin 2018;250:54-9.

33. Eltawaty S, Almagboul AZ, Mohammad AEH, Bufarwa SM. The potential antioxidant and hepatotoxicity of methanolic extract of leaves of libyan capparis spinosa subsp orientalis (duh.) jafri in rats. World Journal of Pharmaceutical Research. 2018;(October).

34. El-waseif M, Saed B. Using Egyptian Caper Seeds Oil (Capparis spinosa L) as a Natural Antioxidant to Improving Oxidative Stability of Frying Oils Using Egyptian Caper Seeds Oil (Capparis spinosa L) as a Natural Antioxidant. World Journal of Dairy & Food Sciences. 2018;(January).

35. Tlili N, Feriani A, Saadoui E, Nasri N, Khaldi A. Capparis spinosa leaves extract: Source of bioantioxidants with nephroprotective and hepatoprotective effects. Biomedicine and Pharmacotherapy. 2017;87:171-9.

36. Yarizade A, Niazi A, Kumleh HH. Investigation of Antiglycation and Antioxidant Potential of Some Antidiabetic Medicinal Plants. Journal of pharmaceutical sciences and research. 2017;2019(January 2018).

37. Mazarei F, Jooyandeh H, Noshad M, Hojjati M. Polysaccharide of caper (Capparis spinosa L.) Leaf: Extraction optimization, antioxidant potential and antimicrobial activity. International Journal of Biological Macromolecules. 1 févr 2017;95:224-31.

38. Dibas JI, Yaghi BM, Mansi IA, Mhaidat NM, Al-Abrouni KF. Biological and Chemical Sciences Screening for Cytotoxic and Antioxidant Activity of Selected Wild Plants at Shafa Badran, Amman, Jordan. Research Journal of Pharmaceutical. 2017;8(489):489-97.

39. Aichi-Yousfi H, Meddeb E, Rouissi W, Hamrouni, L, Rouz S, Rejeb MN, et al. Phenolic composition and antioxidant activity of aqueous and ethanolic leaf extracts of six Tunisian species of genus Capparis – Capparaceae. Industrial Crops and Products,. 92:218-26.

40. Mansour RB, Jilani IBH, Bouaziz M, Gargouri B, Elloumi N, Attia H, et al. Phenolic contents and antioxidant activity of ethanolic extract of Capparis spinosa. Cytotechnology. 2016;68(1):135-42.

41. Al-janabi NMS. Assay plant as natural antioxidant of capparis spinosa root bark. International Journal of Current Research In Chemistry and Pharmaceutical Sciences. 2015;2:62-8.

42. Tlili N, Mejri H, Anouer F, Saadaoui E. Phenolic profile and antioxidant activity of Capparis spinosa seeds harvested from different wild habitats. Industrial Crops & Products. 2015;76:930-5.

43. Aliyazicioglu R, Eyupoglu OE, Sahin H, Yildiz O, Baltas N. Phenolic components , antioxidant activity , and mineral analysis of Capparis spinosa L. African Journal of Biotechnology. 2013;12(47):6643-9.
